# Embedding rapid reviews in health policy and systems decision-making: Impacts and lessons learned from four low- and middle-income countries

**DOI:** 10.1186/s12961-023-00992-w

**Published:** 2023-06-06

**Authors:** Reid C. Robson, Sonia M. Thomas, Étienne V. Langlois, Rhona Mijumbi, Ismael Kawooya, Jesmin Antony, Melissa Courvoisier, Krystle Amog, Robert Marten, Ivdity Chikovani, Devaki Nambiar, Rajani R. Ved, Soumyadeep Bhaumik, Nur Zahirah Balqis-Ali, Sondi Sararaks, Shakirah Md. Sharif, Rugare Abigail Kangwende, Ronald Munatsi, Sharon E. Straus, Andrea C. Tricco

**Affiliations:** 1 Knowledge Translation Program, Li Ka Shing Knowledge Institute, St. Michael’s Hospital, Unity Health Toronto, 209 Victoria Street, 7th Floor, East Building, Toronto, ON M5B 1T8 Canada; 2 Partnership for Maternal, Newborn and Child Health (PMNCH), World Health Organization, Geneva, Switzerland; 3 Alliance for Health Policy and Systems Research, Science Division, World Health Organization (WHO), Geneva, Switzerland; 4 Institute of Health Policy, Management, and Evaluation, Dalla Lana School of Public Health, University of Toronto, Toronto, ON Canada; 5 The Center for Rapid Evidence Synthesis (ACRES), Regional East African Policy Initiative, Uganda Node, Makerere University College of Health Sciences, Kampala, Uganda; 6 Research Department, Curatio International Foundation, Tbilisi, Georgia; 7 The George Institute for Global Health, New Delhi, India; 8 Faculty of Medicine, University of New South Wales, Sydney, Australia; 9 Prasanna School of Public Health, Manipal Academy of Higher Education, Manipal, India; 10 Bill and Melinda Gates Foundation, New Delhi, India; 11 Meta-Research & Evidence Synthesis Unit, The George Institute for Global Health, New Delhi, India; 12 Institute for Health Systems Research, National Institutes of Health, Ministry of Health, Putrajaya, Malaysia; 13 Ministry of Health and Child Care, Harare, Zimbabwe; 14 Zimbabwe Evidence-Informed Policy Network (ZeipNET), Harare, Zimbabwe; 15 Department of Geriatric Medicine, University of Toronto, Toronto, ON Canada; 16 Epidemiology Division and Institute for Health, Management, and Evaluation, Dalla Lana School of Public Health, University of Toronto, Toronto, ON Canada

**Keywords:** Capacity-building, Low- and middle-income economy countries, Knowledge synthesis, Policy decision-making, Rapid reviews

## Abstract

**Background:**

Demand for rapid evidence-based syntheses to inform health policy and systems decision-making has increased worldwide, including in low- and middle-income countries (LMICs). To promote use of rapid syntheses in LMICs, the WHO’s Alliance for Health Policy and Systems Research (AHPSR) created the Embedding Rapid Reviews in Health Systems Decision-Making (ERA) Initiative. Following a call for proposals, four LMICs were selected (Georgia, India, Malaysia and Zimbabwe) and supported for 1 year to embed rapid response platforms within a public institution with a health policy or systems decision-making mandate.

**Methods:**

While the selected platforms had experience in health policy and systems research and evidence syntheses, platforms were less confident conducting rapid evidence syntheses. A technical assistance centre (TAC) was created from the outset to develop and lead a capacity-strengthening program for rapid syntheses, tailored to the platforms based on their original proposals and needs as assessed in a baseline questionnaire. The program included training in rapid synthesis methods, as well as generating synthesis demand, engaging knowledge users and ensuring knowledge uptake. Modalities included live training webinars, in-country workshops and support through phone, email and an online platform. LMICs provided regular updates on policy-makers’ requests and the rapid products provided, as well as barriers, facilitators and impacts. Post-initiative, platforms were surveyed.

**Results:**

Platforms provided rapid syntheses across a range of AHPSR themes, and successfully engaged national- and state-level policy-makers. Examples of substantial policy impact were observed, including for COVID-19. Although the post-initiative survey response rate was low, three quarters of those responding felt confident in their ability to conduct a rapid evidence synthesis. Lessons learned coalesced around three themes – the importance of context-specific expertise in conducting reviews, facilitating cross-platform learning, and planning for platform sustainability.

**Conclusions:**

The ERA initiative successfully established rapid response platforms in four LMICs. The short timeframe limited the number of rapid products produced, but there were examples of substantial impact and growing demand. We emphasize that LMICs can and should be involved not only in identifying and articulating needs but as co-designers in their own capacity-strengthening programs. More time is required to assess whether these platforms will be sustained for the long-term.

**Supplementary Information:**

The online version contains supplementary material available at 10.1186/s12961-023-00992-w.

## Background

As many low- and middle-income countries (LMICs) demonstrate commitment towards universal health coverage, there is an increasing demand for evidence to inform health policies and improve health systems. Generation of such evidence requires research valued and prioritized by health systems policy-makers [1], and contextualized to local health system settings [2]. As health systems decision-makers seek to address urgent policy and systems needs – coronavirus disease 2019 (COVID-19) mitigation efforts, for example – one of the most frequently voiced requests is for evidence in the form of a so-called rapid review [3, 4]. Rapid reviews are accelerated evidence syntheses offering several advantages compared with systematic reviews that can provide decision-makers with relevant and actionable evidence [5, 6].

In 2017, the WHO’s Alliance for Health Policy and Systems Research (AHPSR) partnership published Rapid reviews to strengthen health policy and systems: A practical guide [7] for conducting rapid reviews to strengthen health policy and systems, with a focus on LMICs. The guide recognized the need for rapid reviews in public health emergencies, such as infectious disease outbreaks in which health systems are pressured for a rapid response (like COVID-19), and in various routine situations in which informed decisions about health systems must be made quickly.

Rapid reviews need to include not only experts in review methods but also local experts who understand the cultural, social, economic and political contexts. Local experts can clarify the review questions and contextualize any recommendations based on the evidence synthesized. Local "ownership" of research is also a catalyst for uptake, especially in the health policy and systems research (HPSR) context (2). However, accessing local LMIC expertise, amongst health system actors including policy-makers, service users and HPSR researchers, may be a challenge amidst a turbulent political or economic environment or where funding may be limited. Recognizing these challenges, the AHPSR sought to strengthen LMIC capacity by creating “rapid response platforms” through the Embedding Rapid Reviews in Health Systems Decision-Making (ERA) initiative. This report describes the ERA initiative and its impacts.

To facilitate institutionalization from the outset, the ERA initiative sought to support platform teams already embedded in a public institution with a health policy or systems decision-making mandate. The decision-making institution had to provide complementary in-kind or financial support. In addition, platform teams needed to have relevant expertise related to evidence synthesis and rapid reviews, and experience producing health policy and systems research (HPSR) syntheses in response to requests by policy- and decision-makers, including use of rapid products (in the Rapid Review Guide, three different types of rapid evidence synthesis products are highlighted: rapid inventories, rapid response briefs and rapid reviews) [7]. Funding of up to US$ 200 000 per platform was available, with activities to be implemented between July 2018 and November 2019. LMICs were defined using the 2017 World Bank classification [8].

A call for proposals to establish four ERA platforms in LMICs opened in April 2018, and 16 submissions from 14 LMICs were received. Submissions were peer-reviewed by an experienced panel independent of the Alliance. At the end of this process, in June of 2018, an adjudication panel selected ERA platforms in Georgia, India, Malaysia and Zimbabwe.

The purpose of this paper is to: (1) describe the establishment of the ERA platforms in these four LMICs, including challenges and lessons learned, and (2) evaluate the success of the ERA initiative, in particular the effects on policy development and health systems arrangements.

## Methods

### The technical assistance centre

Prior to the selection of the four LMICs, a technical assistance centre (TAC) was established, with a mandate to develop and implement a capacity-strengthening scheme to support the establishment of the rapid response platforms and provide technical advice. The TAC established the eligibility criteria and oversaw the peer review process for the selection of the LMICs. The TAC was also established through a proposal submission and peer review process and had to hold expertise in both rapid review methods and rapid response services. The selected TAC was a collaboration between the Knowledge Translation Program at St. Michael’s Hospital of Unity Health Toronto in Canada and The Center for Rapid Evidence Synthesis (ACRES) at Makerere University College of Health Sciences in Kampala, Uganda. The TAC consisted of experts with substantial experience in evidence synthesis, rapid reviews, knowledge user engagement, adult education, and health policy and systems research.

### Inception workshop

In July of 2018, the TAC hosted a 3-day inception workshop in Kuala Lumpur, Malaysia, with 13 representatives from the four platforms, and included at least one policy-maker and one researcher from each LMIC (Additional file 1: Appendix 1). Five advisors from the Toronto and Uganda TAC teams and the AHPSR acted as facilitators (SMT, EVL, RM, IK and ACT) and presented training modules on the use of rapid evidence syntheses in health policy and systems decision-making and engaging policy-makers for knowledge uptake. Each platform presented expanded versions of their original submitted proposals for establishing a platform and received feedback from the group.

### Needs assessment of participating LMICs

ERA platforms required a complex set of skills in evidence synthesis, rapid review methods and policy-maker engagement applied to health policy and systems decision-making. This necessitated a capacity strengthening scheme aligned with the needs of the platforms. A needs assessment was conducted following the inception workshop and performance gaps determined in relation to the desired outcomes of the initiative using online surveys in Qualtrics to assess participants’ knowledge, skills and self-efficacy [9, 10]. Survey links were sent to the leads of each platform to circulate to team members who would be involved in the initiative, including researchers and policy-makers [11].

The survey included multiple choice and open-ended questions on participants’ self-identified knowledge, experience and confidence in synthesis of health policy and systems evidence and rapid reviews, as well as learning priorities (Additional file 1: Appendix 2), and was followed by interviews with platform leads to interpret the results. Results were used to identify content relevant for each platform, the preferred modality of delivery and the support needed to establish rapid response platforms in their respective countries.

**Fig. 1.**
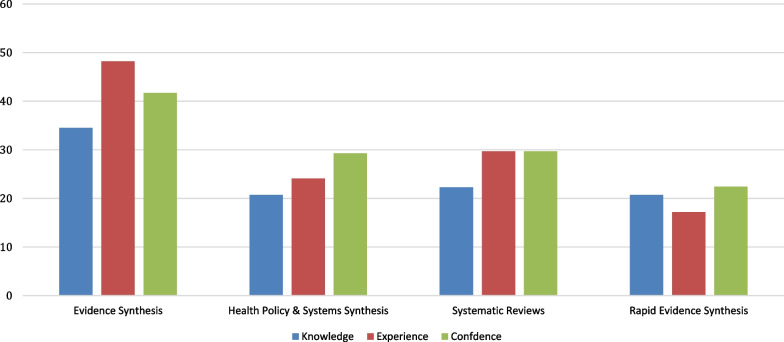
Needs assessment results – % that agree/strongly agree

Overall, 58 participants, mainly researchers and some policy-makers – Georgia (*n* = 14), India (*n* = 1), Malaysia (*n* = 32) and Zimbabwe (*n* = 11) – participated in the initial needs assessment survey (Additional file 1: Appendix 3). Knowledge, experience and confidence were highest for “evidence synthesis”, where 41.7% felt confident (“agree” or “strongly agree”) in their ability to synthesize evidence but were lower for “synthesizing health policy and systems evidence” (29.3%) and “conducting systematic reviews” (29.7%). Scores were lowest for “conducting rapid evidence synthesis”, where only 22% (13 of 58) were confident in their ability to conduct a rapid evidence synthesis (Fig. 1). One country scored somewhat higher than the others for confidence with evidence synthesis and systematic reviews.

### Capacity-strengthening components

**Fig. 2:**
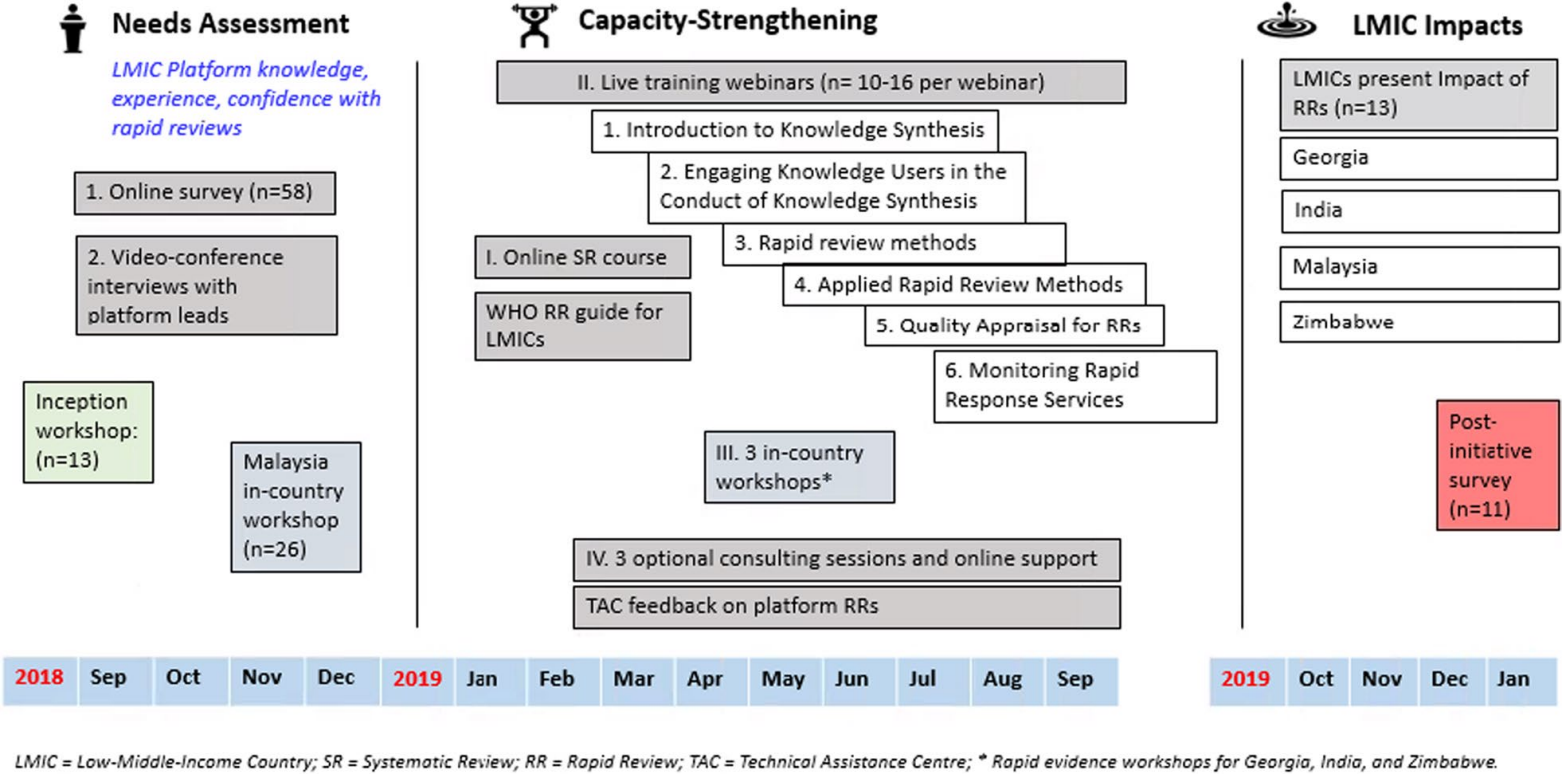
Embedding Rapid Reviews (ERA) overview

The TAC used the needs assessment to design the capacity-strengthening program. The key components are described below and summarized in Fig. 2.

#### Introductory online systematic reviews course

To address gaps in knowledge, experience and confidence with evidence synthesis methods, the TAC provided access to an asynchronous introductory online self-paced course on systematic review methods [12], as well as a rapid review methodology guide [7, 13]. The capacity-strengthening modalities were selected to provide researchers with varying levels of knowledge and experience opportunity to gain confidence in evidence synthesis. The approach was interactive and provided opportunities to produce knowledge while building skills. The number of attendees and those completing the course were recorded.

#### Live training webinars

A series of live training webinars were developed to cover the conduct of rapid reviews and production of rapid evidence products. The webinar topics were informed by the chapters of the *Rapid reviews to strengthen health policy and systems: A practical guide* [7], discussions at the inception workshop and results of the needs assessment. Building on the online course, six live webinars were delivered to the four LMICs, and attended jointly between February and September of 2019 (Fig. 1). Topics were designed to progressively build researchers’ knowledge [10, 14], and platforms were encouraged to use an integrated knowledge translation approach [15] with knowledge users during the conduct of their rapid evidence syntheses [16]. Participants completed online evaluation surveys, and webinars were revised iteratively based on feedback (Additional file 1: Appendix 4). The online course and webinars were directed towards researchers and were optional for policy-makers.

#### In-country workshops

A 3-day in-person workshop on rapid evidence synthesis methods and use in policy and systems decision-making was provided by the TAC in each of the four countries and included ERA researchers as well as policy-makers. The first session was in held Malaysia in November 2018, with 26 participants from three universities and five departments from the Ministry of Health. The remaining workshops were held in May and June of 2019, at the mid-point of the ERA initiative in Georgia (*n* = 22), India (*n* = 17) and Zimbabwe (*n* = 16). A sample workshop agenda may be found in Additional file 1: Appendix 5. Participant feedback was collected with an online survey after each workshop.

#### Platform consultations and online support

The TAC offered three consultation sessions with each of the platform leads over the duration of the initiative to discuss platform formation progress, identify challenges and successes, and provide feedback on the rapid products in development. A sample consultation agenda may be found in Additional file 1: Appendix 6. In addition, over the course of the initiative, the TAC supported platforms via email and the CANVAS online discussion board [17].

### Impacts and evaluation for the entire ERA initiative

#### Impacts on health policy and systems

During the final webinar in October 2019, platforms presented their efforts towards generating demand for rapid reviews, and the rapid products developed through the ERA program along with their impact on decision-making (specifically, they were asked to record the “impact of rapid product on health policy-making and health system strengthening; how was the product used”). Monthly updates were also collected for each platform throughout 2019 and in May 2020 to monitor progress and document details regarding demand generation, knowledge user engagement, topics addressed, any barriers and facilitators encountered and the impact of each rapid product. A sample monitoring form may be found in Additional file 1: Appendix 7. Based on the webinar platform presentations and the monthly updates, a single reviewer summarized the knowledge user request, the product delivered, its turn-around time, noteworthy challenges for review production, and significant impacts and downstream goals. A second reviewer validated the summary.

#### WHO ERA capacity building platform evaluation

The impact of the initiative on researchers’ knowledge, experience and confidence was evaluated using a post-initiative online survey in January 2020 and mirrored the initial needs assessment survey. The target audience included the producers of the rapid evidence syntheses and decision-makers. The final feedback survey included 31 questions on the overall impact of the ERA initiative and 38 questions related to the process of implementing the initiative (Additional file 1: Appendix 8).

#### TAC assessment of the ERA initiative – challenges and lessons learned

Key challenges and lessons learned were identified and described by the TAC.

## Results

### LMIC platform participants

All LMICs developed their platforms based on existing embedded teams and used funds to augment expertise in alignment with their strategies and to produce the rapid evidence syntheses. The ERA platform in Georgia expanded their parliament’s research department and engaged an outside expert nongovernmental organization (NGO). The team in Zimbabwe similarly used an expert NGO to lead and expand a limited internal evidence-generating capacity. India’s ERA platform used a central national government committee and an outside agency to connect with a broad range of federal- and state-level decision-making institutions with varied experience using evidence syntheses. The team in Malaysia embedded their platform within an institute experienced in providing evidence for various ministry decision-makers.

The number of core LMIC platform researchers was inferred from the LMIC monitoring reports and ranged from 6 to 7: Georgia (*n* = 7), Malaysia (*n* = 6), India (*n* = 6) and Zimbabwe (*n* = 7), with additional support from external collaborators depending on the project (Additional file 2: Table S1). Except for Malaysia, who launched their platform much earlier than the other countries, the first live training webinar began before platforms had their teams fully in place (Additional file 2: Table S1B). Teams were in place soon after though, followed by in-country workshops (led by the TAC), and initiation of the first rapid reviews (while India’s first review began prior to the workshop). Webinars continued as the rapid reviews progressed.

### Rapid synthesis outputs

**Fig. 3:**
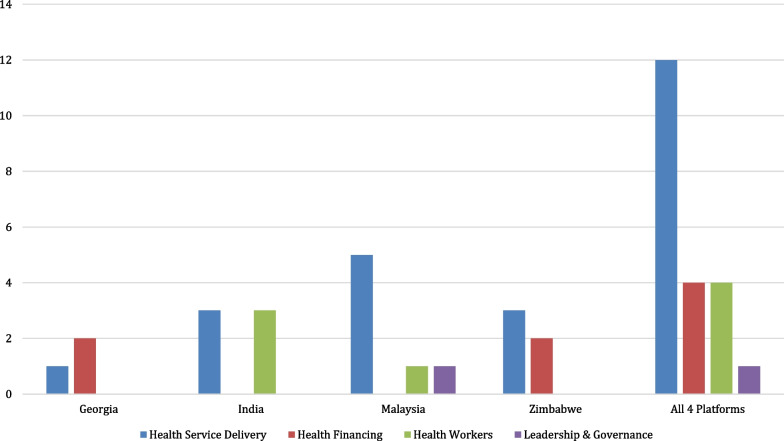
Platform Rapid Review Outputs

The Inception Workshop took place in July of 2018, whereby platforms began implementing their strategic plans, building their teams and connecting with policy-makers to discuss the process of stimulating and sustaining demand. Work on the first rapid products began midway through 2019 (except for Malaysia whose first review began at the end of 2018) with AHPSR support to the end by November. Changes in political climates in some countries caused substantial staffing delays, and as such, a no-cost extension was provided to May of 2020 to allow platforms to continue to receive technical support and complete their rapid products. All platforms encountered barriers, including disruptions because of COVID-19, but nonetheless made progress completing a total of 21 rapid reviews (including COVID-19 support) in response to end-user HPSR requests. The primary focus of the rapid syntheses was in the area of health service delivery (59%), followed by health system financing (18%) and health workforce (18%), and leadership and governance (5%; Additional file 2: Table S2; Fig. 3, based on the WHO health systems building blocks framework) [18]. No requests were observed in the areas of health information systems or access to essential medicines.

### Barriers and facilitators

Barriers to knowledge user engagement in the conduct of rapid syntheses were indentified in half of the 21 reviews listed in Additional file 2: Table S3. By far the most common barriers were those arising due to constraints of the policy-maker (their time, attendance or attention), seen in about half the reported cases. A second theme centred on core team research capabilities (lack of experience or expertise, retention of staff, insufficient time up front to finalize the research question and challenges related to explaining complex findings).

Facilitators of knowledge user engagement were provided in just over half the reviews (Additional file 2: Table S3). High-level support from decision-makers (government interest, cabinet agreement, topic awareness and director support) was the most commonly identified theme. Relationship-building with the right knowledge users (through early stakeholder mapping, ensuring shared goals and engaging continuously) was a second theme. Thirdly, technical facilitators included establishing agreement on the research question, inclusion criteria and search strategy; use of grey literature; and expertise in communicating the findings (use of frameworks). Finally, administrative enablers included negotiated timelines; use of phone, email and virtual meetings; and in one case, a non-disclosure agreement.

Platforms used various supporting products introduced in the ERA training, including protocols, scoping reviews and patient/population, intervention, comparison and outcomes (PICO) documents, as they worked with knowledge users on the reviews. Additionally, in-country workshops introduced a rapid response service toolkit aligned with the steps described in the WHO’s Evidence-Informed Policy-making Network (EVIPNet) framework [19].

### Impacts on health policy and systems

Platform monthly updates provided evidence of knowledge user uptake of rapid syntheses, and impacts where known. Knowledge users were typically engaged for discussion and refinement of the research questions, and to some degree for literature search strategies and review of preliminary results. Examples from each of the LMIC platforms are highlighted below. Impacts were more readily evident for COVID-19-related reviews than for the reviews started in 2019, several of which were significantly delayed because of the COVID-19 pandemic (Additional file 2: Table S3; Additional file 1: Appendices 9, 10).

#### Georgia

The ERA platform in Georgia was a collaboration between the Curatio International Foundation (CIF; a non-profit NGO with a mission to improve health through better functioning health systems) and a policy-making body within the Parliament of Georgia – the Health and Social Issues Committee (HSIC). A presidential election and a parliament research department reorganization slowed establishment of the Georgia platform. The initial platform consisted of three experts from the CIF, and four staff from Parliament’s research department. The HSIC Secretariat held introductory meetings with parliamentary managers, policy heads at the Ministry of Health and Social Affairs and leaders at the National Centre for Disease Control. Efforts to generate demand were maintained through a policy dialogue in 2019, directed at policy-makers within Georgia’s health system. Research questions arose from within the HSIC and from the Ministry of Internally Displaced Persons from the Occupied Territories, Labour, Health and Social Affairs (MoILHSA).

The ERA-Georgia platform responded to two (non-COVID-19) requests during the initiative. Highlighting one example, the HSIC commissioned a rapid response product to examine how pharmaceutical pricing and purchasing policies influence population access to pharmaceuticals. The review was produced in 6 weeks and synthesized evidence on mechanisms to enable access as well as implementation considerations. External reference pricing was a key recommendation, and the review informed an HSIC working group, which also requested CIF participation, and eventually issued a set of recommendations including reference pricing as a critical component to pharmaceutical access (Additional file 2: Table S2; Additional file 1: Appendix 9).

At the beginning of the COVID-19 pandemic, the MoILHSA issued an urgent request to synthesize best practice for pandemic mitigation. CIF researchers produced a review on how countries “flattened the curve”, developed caseload models based on social distancing and provided a set of policy recommendations (Additional file 2: Table S2; Additional file 1: Appendix 10). The review was delivered to the MoILHSA and the Prime Minister’s office. Findings were widely disseminated and sparked discussions on social media and TV. Additional COVID-19 requests followed, and rapid responses included reviews and recommendations for the introduction and lifting of containment measures, financing models for COVID-19 hospital case management, and telemedicine provider payment mechanisms.

#### India

The Embedded Rapid Evidence Syntheses Unit (ERESU), at The George Institute for Global Health (TGI), New Delhi, was embedded within the National Health Systems Resource Centre (NHSRC), in the Ministry of Health & Family Welfare (MoHFW). Recruitment of staff to conduct the reviews, overseen by TGI experts, was challenging and continued throughout 2019. To generate demand, the ERESU created a calendar to track events where the ERESU’s services could be marketed. In one case, flyers were distributed at a national consultative meeting with over 100 program managers in attendance from over a dozen states.

The ERESU responded to five non-COVID-19 requests during the initiative (Additional file 2: Table S2; Additional file 1: Appendix 9). In one example, the State Health Systems Resource Centre (SHSRC) in Madhya Pradesh, was interested in policy on the use and cost–effectiveness of angle of tri-radius (ATD) measurement as a breast cancer screening tool in women in resource-poor settings. This approach was perceived as cost–effective, non-invasive and accessible. The rapid review took 7 weeks to complete, and a policy brief and supplement were provided. The brief contained policy recommendations, and a recommendation for a pilot study on the diagnostic accuracy of the ATD measurement.

For COVID-19, the National Health Systems Resource Centre requested support for the planning and development of resources to ensure preparedness of community front-line health workers (FLHWs). The ERESU produced a rapid evidence synthesis in 3 days (Additional file 2: Table S2; Additional file 1: Appendix 10). A policy brief was provided, and its findings were widely adopted, including a MoHFW brochure for India’s FLHWs, policy for the state of Odisha regarding field surveillance, a preparedness checklist for rural community settings sponsored by a collaboration of clinicians and public health researchers from leading institutions in India, and use by many other organizations and advocacy groups.

#### Malaysia

The ERA platform in Malaysia was embedded in the Institute for Health Systems Research (IHSR) in the Ministry of Health, and is referred to as the Malaysian Alliance for Embedding Rapid Reviews in Health Systems Decision-Making (MAera). MAera was established early on, at the end of 2018, with six core staff, and five policy-maker champions from Ministry programs. A high staff turnover necessitated a constant recruitment and onboarding process for research assistants. MAera launched an extensive plan for generating demand that included engagement with a variety of groups, which resulted in several requests. Six non-COVID-19 rapid reviews were completed and are described in Additional file 2: Table S2 and Additional file 1: Appendix 9. MAera’s first request was from the Family Health Development Division, Ministry of Health Malaysia (BPKK), regarding the required competency/qualification for healthcare practitioners managing antenatal cases in primary care. The initial focus was on preparing midwives to meet the increasing demands of complex maternal cases. The review took 5 months and found that most countries with better maternal mortality rates had midwives with degree-level education. Directors (knowledge users) used the report to argue for continued support of the previously approved plan of piloting degree-level education for nurses. MAera’s reviews took between 10 days and 10 months. MAera also produced an online public dashboard of COVID-19 government responses (Additional file 2: Table S2 and Additional file 1: Appendix 10).

#### Zimbabwe

The Zimbabwe Evidence-Informed Policy Network (ZeipNET), a non-governmental organization working in Zimbabwe to promote the use of research evidence, partnered with the Ministry of Health and Child Care (MoHCC) to embed a platform for rapid reviews within the Ministry, named the “Embedding Rapid Reviews in Health Systems Decision-Making in Zimbabwe” (ERAZ) project. ERAZ team core members comprised seven MoHCC staff with supporting expertise from ZeipNET and the Zimbabwe College of Public Health Physicians (ZCPHP) who were recruited as needed. The MoHCC staff conducted ERAZ projects in addition to their regular MoHCC work. Initial sensitization meetings were held by ERAZ staff, directed at Ministry senior managers, as well as leaders from the National Institute of Health Research. Demand generation continued through 2019, executing a plan for ongoing sensitization meetings, and evolving plans for knowledge cafes, policy dialogues and an ERAZ website.

Three non-COVID-19 rapid reviews were completed during the initiative and are described in Additional file 2: Table S2 and Additional file 1: Appendix 9. ERAZ also provided rapid reviews in response to two high-level requests regarding COVID-19 (Additional file 2: Table S2, Additional file 1 Appendix 10). One rapid review examined institutional quarantine for returning citizens and influenced the country’s policy and guidelines. The review was subsequently updated in response to a request for evidence on the recommended duration of quarantine, and evidence was provided at the request of the Office of the President and Cabinet to guide revision of the national policy on COVID-19-related quarantine. The findings from another rapid review on the public use of face masks were adopted by the National COVID-19 response team. Subsequently, the Cabinet introduced a policy mandating face masks in public settings. In both cases, ERAZ completed the reviews in about 2 weeks.

### Participant evaluation of the entire ERA initiative

#### Impact of the initiative on researcher’s knowledge, skills and confidence

Overall, 21 individuals – Georgia (*n* = 5), India (*n* = 4), Malaysia (*n* = 7) and Zimbabwe (*n* = 5) – across the four LMIC platforms participated in the ERA initiative through one or more of the six live training webinars. However, only 11 participants (individual platform counts omitted for confidentiality) completed the online post-initiative evaluation survey (Additional file 1: Appendix 11). The 11 participants rated the sessions as good or important, with an average score of 4.4 out of 5. All items assessed were considered positive, with the lowest average item score being 4.3. All participants indicated they would recommend the initiative. Interactive components, hearing candid experience from presenters, and hands-on opportunities to apply content were most appreciated. Areas for improvement included offering longer in-person workshops than the 3 days provided, more opportunities to apply content, and including policy-makers as presenters. In total, 8 of 11 (73%) agreed or strongly agreed that they felt confident in their ability to conduct rapid evidence syntheses.

### Researcher assessment of knowledge user engagement

About half (5 of 11) of respondents felt confident that they could stimulate demand for rapid evidence synthesis products, and that they could negotiate a rapid review question (5 of 11; Additional file 1: Appendix 11). About 73% (8 of 11) felt confident in their ability to engage with the end-user, while 82% indicated that they had experienced barriers when conducting rapid evidence synthesis or engaging with knowledge users. The most common challenge reported was that of finalizing the question with the end-user. Other concerns cited government bureaucracy and political effects, balancing quality with urgency and policy-maker availability during the review process, as well as that of ERA team members. Participants generally felt the ERA initiative increased demand for rapid evidence synthesis products as well as their uptake.

### TAC assessment of the ERA Initiative: Challenges and lessons learned

Key challenges and lessons learned were identified by the TAC along three key themes: the importance of context-specific expertise, cross-platform learning, and planning for sustainability (Additional file 2: Table S4). Context-specific expertise is important because the gathering of HPSR evidence in a LMIC setting is complex. Platforms might have benefited from additional support from HPSR experts based in other LMIC settings. More opportunities for deeper cross-platform learning were also desired, and suggestions were proposed, including both formal and informal approaches. Finally, sustainability of the platforms was of paramount concern. Given the success of inception start-up plans, the TAC recommended similar plans for platform sustainability. See Additional file 2: Table S3 for further details. While the TAC maintained steady support to the platforms, it did not directly facilitate the in-country processes of generating demand, engagement or uptake.

Participant evaluations for the capacity-strengthening components for the online systematic review course, the live webinars and in-country workshops, as well as a summary of platform consultations and online support, can be found in Additional file 1: Appendix 12.

## Discussion

This paper described the implementation of the ERA capacity-strengthening program funded by the WHO AHPSR for the establishment of rapid review platforms in four LMICs. Support was initially provided for 1 year, and tracking continued for an additional 6 months proximate to the onset of the global COVID-19 pandemic. The capacity-strengthening program was attuned to platform-specific needs, and consisted of a set of live webinars, in-country workshops and consultative support, all overseen by a central technical assistance centre. Platforms produced over 20 rapid syntheses at policy-maker request, including syntheses for COVID-19. Most syntheses included policy-maker participation. Nearly three quarters of those responding post-initiative felt confident in their ability to conduct a rapid evidence synthesis. Pre-initiative, this was about one quarter (but with a much larger sample). Significant impacts were recorded in several cases, while insufficient time had elapsed to observe impact in others. Most syntheses requested were relevant to national- and state-level policy.

Rapid reports were most common in the areas of health service delivery, health financing and healthcare workers. In a recent WHO Alliance report on the pilot of rapid reviews in three different LMICs, health service delivery was also prominent, receiving the most questions, followed by health technology assessment (HTA) and clinical questions (considered part of health service delivery in our analysis).

The no-cost extension accommodated significant staffing delays for some platforms due to changes in political climates, allowing platforms to continue to receive technical support and complete their rapid products. The extension also overlapped with the start of the COVID-19 pandemic, which caused some attention to shift from the original reviews to COVID-19 HPSR questions. The original time horizon was short regardless, approximately 1 year, and this limited full reporting of impact. Future programs may want to support and track for a minimum of 2 years, and possibly longer to ensure platform sustainability.

Nonetheless, the no-cost extension allowed observation of substantial impact for the original HPSR requests and also for COVID-19 health policy. India’s rapid review recommendations on preparing FLHWs for COVID-19, for example, had national and international uptake and is now highlighted on the WHO Alliance’s website [20]. India’s initial FLHWs rapid review was prepared in just 3 days, responding to not only an urgent but a rapidly evolving situation. Recently, the distinction between “emergency” and “rapid” modes of reviewing has been introduced, noting that rapid review standards may need to be tailored further for emergency contexts [21]. Collection of observable differences between these review approaches may be of interest for future capacity-strengthening programs.

Perhaps not surprisingly, barriers and facilitators to knowledge user engagement also seemed heavily dependent on the knowledge user’s situation – in some cases, affecting their availability to participate beyond the initial launch of a review – and the technical expertise available. This reinforces the importance of an evidence-driven strategy for knowledge user engagement, based on the LMIC setting, and ensuring that technical advisory and support is available and accessible.

As only 11 research participants completed the online post-initiative evaluation survey, it remains unknown whether those not completing the survey were similarly satisfied with the ERA initiative. This could also reflect staff turnover in these platform countries, which was one challenge implementing this initiative. Future initiatives should pursue higher response rates to gain a full view of their impact. Similarly, feedback from the live training sessions needs to be considered in light of those who did not respond.

Lessons learned had themes of providing sufficient context-specific expertise, enabling cross-platform learning, and, of paramount importance, planning for sustainability. Successful policy-maker engagement in particular is essential to ensure platform sustainability (as well as a useful product). Policy-maker engagement is, however, an iterative process that consumes time and resources and was observed to lengthen the review process in several cases. Nonetheless, policy-maker engagement is necessary to ensure that review findings are policy-relevant and uptake maximized. As such, we emphasize routine policy-maker engagement carefully monitored within the context of a platform sustainability plan. The reader is referred to the WHO’s Methods Guide [13] for further discussion.

The ERA initiative was designed by the TAC and tailored to the four platforms’ needs assessments. Future initiatives may consider using a co-design process that partners with participants not only for needs assessment but also in educational design, such as using local policy-makers to lead training webinars. This should facilitate proactive responses to country-specific barriers attuned to each platform’s unique setting. Co-designing the initiative can facilitate greater participant support, who as co-designers are not merely recipients of the initiative but rather are co-creating the very initiative that will support their own capabilities development. Based on participant feedback, it is important to ensure sufficient time for program design is allotted up front.

## Conclusions

The ERA initiative successfully established rapid response platforms in four LMICs. Platforms provided rapid syntheses across a range of HPSR themes, and successfully engaged national- and state-level policy-makers, generating additional examples of significant policy impacts involving COVID-19. More time is required to assess whether these platforms will be sustained by the LMICs, and we recommend future initiatives encompass a longer horizon. Lessons learned included the importance of providing sufficient context-specific expertise, facilitating cross-platform learning and planning for sustainability. LMICs can and should be involved not only in identifying and articulating needs but also as co-designers in their own capacity-strengthening programs to ensure context-relevant considerations and participant support.

## Supplementary Information

**Additional file 1. **Appendices 1–12.

**Additional file 2.** Tables S1–4.

## Data Availability

The datasets used and/or analysed during the current study are available from the corresponding author on reasonable request.

## Abbreviations

ACRES: The Center for Rapid Evidence Synthesis
AHPSR: Alliance for Health Policy and Systems Research
ATD: Angle of Tri-radius
BPKK: Family Health Development Division, Ministry of Health Malaysia
CIF: Curatio International Foundation
ERA: Embedding Rapid Reviews in Health Systems Decision-Making
ERAZ: Embedding Rapid Reviews in Health Systems Decision Making in Zimbabwe
ERESU: Embedded Rapid Evidence Syntheses Unit
EVIPNet: Evidence-Informed Policy-making Network
FLHW: Front-line health worker
HPSR: Health policy and systems research
HSIC: Health and Social Issues Committee
HTA: Health Technology Assessment
LMIC: Low- and middle-income countries
MAera: Malaysian Alliance for Embedding Rapid Reviews in Health Systems Decision-Making
MoHCC: Ministry of Health and Child Care
MoHFW: Ministry of Health & Family Welfare
MoILHSA: Ministry of Internally Displaced Persons from the Occupied Territories, Labour, Health and Social Affairs
NHSRC: National Health Systems Resource Centre
PICO: Patient/population, intervention, comparison and outcomes
SHSRC: State Health Systems Resource Centre
TAC: Technical assistance centre
ZCPHP: Zimbabwe College of Public Health Physicians
ZeipNET: Zimbabwe Evidence-Informed Policy Network
