# Supplementary material for: Embedding rapid reviews in health policy and systems decision-making: Impacts and lessons learned from four low- and middle-income countries
Source: Health Res Policy Syst. 2023 Jun 6;21:45. doi: 10.1186/s12961-023-00992-w (PMC10243686; doi:10.1186/s12961-023-00992-w)
Supplement: Supplementary file 1 — Additional file 1. Appendices 1–12. [file 12961_2023_992_MOESM1_ESM.docx]

**Appendices**

| [Appendix 1: Inception Workshop Agenda 1](#_Toc115876649)  [Appendix 2: Needs Assessment Survey 4](#_Toc115876650)  [Appendix 3: Needs Assessment Survey Results 7](#_Toc115876651)  [Appendix 4: Webinar Evaluation Form 44](#_Toc115876652)  [Appendix 5: Sample In-Country Workshop Agenda 48](#_Toc115876653)  [Appendix 6: Sample Consult Call Meeting Agenda 52](#_Toc115876654)  [Appendix 7: Sample of Platform Monitoring Form 54](#_Toc115876655)  [Appendix 8: ERA Evaluation Survey 56](#_Toc115876656)  [Appendix 9: LMIC Platform Outputs 64](#_Toc115876657)  [Appendix 10: LMIC Platform Outputs Related to COVID-19 70](#_Toc115876658)  [Appendix 11: ERA Evaluation Survey Results 73](#_Toc115876659)  [Appendix 12: Evaluation and Feedback on ERA Components 96](#_Toc115876660) |
| --- |

# Appendix 1: Inception Workshop Agenda

| **Time (Duration)** | **Day 1: Tuesday July 17^th^, 2018** | **Advisors** |
| --- | --- | --- |
| **9:00-9:30**  (30 mins) | - Welcome and Introduction | All |
| **9:30-10:00**  (30 mins) | - Evidence-informed health policy and systems decision-making: the role of evidence synthesis | Dr. Etienne Langlois |
| **10:00-10:45**  (45 mins) | - Introduction to rapid reviews and rapid evidence synthesis products | Dr. Andrea Tricco |
| **10:45-11:00 Morning Break & Networking** | | |
| **11:00-12:30**  (90 mins) | - Developing rapid reviews of health policy and systems evidence in LMIC settings | Drs. Rhona Mijumbi & Ismael Kawooya |
| **12:30-13:30 Lunch** | | |
| **13:30-14:15**  (45 mins) | - Stimulating demand and promoting uptake of rapid evidence to support policy and systems decisions | Dr. Rhona Mijumbi |
| **14:15-15:15**  (60 mins) | - *Large Group Discussion:* ERA platforms present work plans - Advisors provide feedback and ask questions | All |
| **15:15-15:30 Afternoon Break & Networking** | | |
| **15:30-16:30**  (60 mins) | - *Large Group Discussion (continued):* ERA platforms present work plans - Advisors provide feedback and ask questions | All |
| **16:30-17:00**  (30 mins) | - Final comments and feedback from advisors | All |

| **Time (Duration)** | **Day 2: Wednesday July 18th, 2018** | **Advisors** |
| --- | --- | --- |
| **9:00-9:30**  (30 mins) | - Introduction to teaching sessions (Sonia)   Session 1:   - Stimulating and sustaining demand - Engaging policy-makers - Receiving and clarifying a policy question | Dr. Etienne Langlois |
| **9:30-10:30**  (60 mins) | Session 2:   - Refining a research question - Selecting and abstracting evidence | Sonia Thomas |
| **10:30-11:15**  (45 mins) | Session 3:   - Assessing the quality of evidence - Summarizing/synthesizing evidence - Disseminating evidence | Dr. Andrea Tricco |
| **11:15-11:30 Morning Break & Networking** | | |
| **11:30-12:15**  (45 mins) | - Introduction to rapid review methods and approaches from the WHO Guide - Overview of key approaches and shortcuts to developing rapid reviews of Health Policy and Systems Research (HPSR) - Summary of upcoming webinars | Dr. Andrea Tricco & Sonia Thomas |
| **12:15-13:15 Lunch** | | |
| **13:15-14:15**  (60 mins) | - Strategies to stimulate uptake of rapid reviews in LMICs - Resources and tools to support implementation of ERA platforms and impact of rapid reviews | Drs. Rhona Mijumbi & Ismael Kawooya |
| **14:15-15:00**  (45 min) | - *Small Group Exercises:*   - SWOT Analysis | All |
| **15:00-15:15 Afternoon Break & Networking** | | |
| **15:15-17:00**  (150 mins) | - *Small Group Exercises (continued):*   - ERA platforms reflect on strategies to enhance the process and impact of ERA platforms   - Strategies to stimulate demand and uptake | All |

| **Time (Duration)** | **Day 3: Thursday July 19th, 2018** | **Advisors** |
| --- | --- | --- |
| **9:00-9:45**  (45 mins) | - Introduction and overview of the in-country technical training sessions - Enhancing the performance of ERA platforms using the knowledge translation framework | Drs. Rhona Mijumbi & Ismael Kawooya |
| **9:45-11:15**  (90 mins) | - *Large Group Discussion:* Challenges of rapid reviews and potential solutions | All |
| **11:15-11:30 Morning Break & Networking** | | |
| **11:30-12:30**  (60 min) | - Working time for ERA platforms to reflect on how they can apply the workshop learnings to their proposals | All |
| **12:30-13:30 Lunch** | | |
| **13:30-15:00**  (90 mins) | - *Large Group Discussion:* ERA Platforms present key strategic changes and improvements to their proposals, with a strong focus on i) decision-maker engagement, ii) generating and sustaining demand and iii) fostering the use of rapid reviews. | All |
| **15:00-15:15 Afternoon Break & Networking** | | |
| **15:15-16:35**  (80 mins) | - *Large Group Discussion (continued):* ERA Platforms present key strategic changes and improvements to their proposals | All |
| **16:35-17:00**  (25 mins) | - Final comments and feedback from advisors - Next steps & monitoring progress and quality - Workshop closes | All |

# Appendix 2: Needs Assessment Survey

As part of the Embedding Rapid Reviews in Health Systems Decision-Making (ERA) initiative, the Technical Assistance Center (TAC) will be developing and delivering monthly webinar sessions tailored to the needs of the ERA platform participants. In order to ensure the webinars are tailored to meet your learning needs, please take a few minutes to complete the needs assessment survey below.

## Participant information questions

1. Please select your ERA platform location:
   1. India
   2. Georgia
   3. Malaysia
   4. Zimbabwe
2. Full name:
3. Role in the ERA platform:

## Healthy policy and evidence synthesis knowledge and experience questions

Please rate your level of agreement with each statement on a scale from 1 = **strongly disagree** to 5 = **strongly agree**.

1. I currently have a high level of knowledge about evidence synthesis
2. I have experience synthesizing evidence
3. I am confident in my ability to synthesize evidence
4. I currently have a high level of knowledge on how to synthesize health policy and systems evidence
5. I have experience synthesizing health policy and systems evidence
6. I am confident in my ability to synthesize health policy and systems evidence
7. I currently have a high level of knowledge on how to conduct systematic reviews
8. I have experience conducting systematic reviews
9. I am confident in my ability to conduct a systematic review

## Rapid evidence synthesis knowledge and experience questions

Please rate your level of agreement with each statement on a scale from 1 = **strongly disagree** to 5 = **strongly agree**.

1. I currently have a high level of knowledge on how to conduct rapid evidence synthesis and/or rapid reviews.
2. I have experience conducting rapid evidence synthesis
3. I am confident in my ability to conduct rapid evidence synthesis
4. Have you completed any rapid evidence synthesis products to date, (e.g. rapid reviews, rapid policy briefs, rapid evidence summaries, rapid inventories, etc.)?
   1. Yes
   2. No

If yes:

- What rapid product(s) did you complete?
- How many rapid products have you completed?
- Who were the stakeholders involved?
- What was your approach in engaging these stakeholders?
- What stage of the review cycle did you engage these stakeholders in?
  - Please note any challenges and successes experienced when working with stakeholders on rapid reviews:
- Within what timeframe were the rapid products completed?
- What challenges (barriers) did you/your organization encounter when developing the rapid products?
- What strengths (facilitators) did you/your organization leverage to develop the rapid products?

If no,

- What challenges (barriers) could you/your organization face when conducting rapid evidence synthesis and developing rapid products?
- What strengths (facilitator) could you/your organization leverage/utilize to help in conducting rapid evidence synthesis and developing rapid products?

## Webinar questions

1. Please rank the topics below in order of importance (i.e., skills needed immediately and/or the team has low knowledge on the topic) by dragging and dropping them. Place the most important topic (i.e., the topic you think your organization needs training on the most) at the top of the list and the least important topic at the bottom of the list.

| **Topic (drag and drop in order of importance)** |
| --- |
| Overview of evidence synthesis methods |
| Systematic review methods |
| The need for rapid reviews and rapid evidence products to inform health policy and systems |
| Rapid review methods |
| How to develop rapid evidence synthesis products |
| How to improve quality and efficiency in selecting, abstracting, and appraising studies for rapid reviews |
| Selecting rapid review methods for complex questions related to health policy and system improvements |
| Stimulating and sustaining demand from policymakers |
| Engaging policy-makers and health systems managers in the conduct of rapid reviews and development of rapid products |
| How to conduct rapid reviews in LMICs |
| Fostering the use of rapid evidence synthesis products in health policy in low- and middle- income countries |
| Reporting and disseminating rapid review findings |
| Improving the uptake of rapid evidence synthesis products |
| Measuring impact of the ERA platform on policy and decision-making/monitoring and evaluation |
| Learning from the experiences of other countries that have successfully implemented similar initiatives |
| Assessing confidence in evidence using GRADE |

1. Are there any additional skills or topics you would like to see addressed as a webinar?

|  |
| --- |

1. Are there any additional comments/feedback you would like to provide?

|  |
| --- |

# Appendix 3: Needs Assessment Survey Results

Contents

[WHO TAC ERA Learning Needs Assessment Summary 7](#_Toc3792111)

[Survey participation 8](#_Toc3792112)

[Summarized survey responses from platforms 8](#_Toc3792113)

[Appendix A 18](#_Toc3792114)

[WHO TAC ERA Program - Learning Needs Assessment - Georgia 18](#_Toc3792115)

[WHO TAC ERA Program - Learning Needs Assessment – India 24](#_Toc3792116)

[WHO TAC ERA Program - Learning Needs Assessment - Malaysia 28](#_Toc3792117)

[WHO TAC ERA Program - Learning Needs Assessment - Zimbabwe 37](#_Toc3792118)

## WHO TAC ERA Learning Needs Assessment Summary

Winter 2019

## Survey participation

| **Platform** | **Number of survey respondents** | **Number of participants in the CBP** |
| --- | --- | --- |
| Georgia | 14 |  |
| India | 1 |  |
| Malaysia | 32 |  |
| Zimbabwe | 11 |  |

## Summarized survey responses from platforms

**Q1. What is your role in the ERA platform?**

Generally, common roles among the platforms are:

- Project/Team lead/manager
- Co-PI
- Researcher
- Human resources
- Policy maker
- Technical support

**Q2. Please rate your level of agreement with each statement on a scale from strongly disagree to strongly agree.**

*Knowledge synthesis*

- - Knowledge: majority neither agree or disagree that they have high level of knowledge
  - Experience: majority agree that they have experience, except majority in Zimbabwe disagree
  - Confidence: majority of Georgia and Zimbabwe neither agree or disagree that they are confident; India and Malaysia agree they are confident

| **Question** | **Georgia** | **India** | **Malaysia** | **Zimbabwe** | **All** | |
| --- | --- | --- | --- | --- | --- | --- |
| I currently have a high level of knowledge about evidence synthesis | 7 – neither a or d  5 – agree  2 - disagree | 1 – neither a or d | 13 – neither a or d  12 - agree  5 – disagree  1 - SD  1 – SA | 5 – neither a or d  4 - disagree  2 – agree | SD  Disagree  Neither  Agree  SA  Total | 1  11  26  19 (32.8%)  1 ( 1.7%)  58 |
| I have experience synthesizing evidence | 7 – agree  5 - neither a or d  2 – disagree | 1 – agree | 14 – agree  8 – neither a or d  8 – disagree  2 – SA | 5 – disagree  4 – agree  2 - neither a or d | SD  Disagree  Neither  Agree  SA  Total | 0  15  15  26 (44.8%)  2 ( 3.4%)  58 |
| I am confident in my ability to synthesize evidence | 7 - neither a or d  5 – agree  2 - disagree | 1 – agree | 14 – agree  11 – neither a or d  6 – disagree  1 – SA | 5 - neither a or d  3 – disagree  3 – agreed | SD  Disagree  Neither  Agree  SA  Total | 0  11  23  23 (40.0%)  1 ( 1.7%)  58 |

*Synthesizing health policy and systems evidence:*

- - Knowledge: majority neither agree or disagree that they have high level of knowledge, except India agrees to having knowledge
  - Experience: majority in Georgia neither agrees or disagrees, India agress, Malaysia and Zimbabwe disagree
  - Confidence: majority neither agree or disagree that they are confident, except India agrees they are confident

| **Question** | **Georgia** | **India** | **Malaysia** | **Zimbabwe** | **All** |  |
| --- | --- | --- | --- | --- | --- | --- |
| I currently have a high level of knowledge on how to synthesize health policy and systems evidence | 8 - neither a or d  3 – disagree  3 - agree | 1 – agree | 11 – neither a or d  11 – disagree  7 – agree  3 – SD | 6 - neither a or d  4 – diasgree  1 - agree | SD  Disagree  Neither  Agree  SA  Total | 3  18  25  12 (20.7%)  0  58 |
| I have experience synthesizing health policy and systems evidence | 6 - neither a or d  5 – agree  3 - disagree | 1 – agree | 15 – disagree  8 – neither a or d  6 – agree  2 – SD  1 – SA | 5 - disagree  4 - neither a or d  1 – strongly disgree  1 - agree | SD  Disagree  Neither  Agree  SA  Total | 3  23  18  13 (22.4%)  1 ( 1.7%)  58 |
| I am confident in my ability to synthesize health policy and systems evidence | 8 - neither a or d  4 – agree  2 - disagree | 1 – agree | 12 – neither a or d  10 – disagree  9 – agree  1 – SD | 5 - neither a or d  3 – agree  2 - disagree | SD  Disagree  Neither  Agree  SA  Total | 1  14  25  17 (29.3%)  0  58 |

*Conducting systematic reviews:*

- - Knowledge: majority neither agree or disagree that they have high level of knowledge, except India agrees to having knowledge
  - Experience: majority in Georgia neither agrees or disagrees, India agress, Malaysia and Zimbabwe disagree
  - Confidence: majority in Georgia and Zimbabwe neither agree or disagree that they are confident; India and Malaysia agree they are confident

| **Question** | **Georgia** | **India** | **Malaysia** | **Zimbabwe** | **All** |  |
| --- | --- | --- | --- | --- | --- | --- |
| I currently have a high level of knowledge on how to conduct systematic reviews | 6 - neither a or d  5 – disagree  1 - strongly disagree  1 - agree | 1 – agree | 15 – neither a or d  7 – agree  7 – disagree  2 – SD  1 – SA | 7 - neither a or d  2 – agree  1 - disagree | SD  Disagree  Neither  Agree  SA  Total | 1  13  28  11 (20.4%)  1 ( 1.9%)  54 |
| I have experience conducting systematic reviews | 5 - neither a or d  4 – disagree  2 – strongly disagree  2 - agree | 1 – agree | 11 – disagree  10 – agree  8 – neither a or d  2 – SD  1 – SA | 4 - disagree  4 - neither agree nor disagree  2 - agree | SD  Disagree  Neither  Agree  SA  Total | 2  19  17  15 (27.8%)  1 ( 1.9%)  54 |
| I am confident in my ability to conduct a systematic review | 5 - neither a or d  3 – disagree  2 – strongly disagree  2 – agree | 1 – agree | 11 – agree  10 – neither a or d  9 – disagree  1 – SD  1 – SA | 6 - neither a or d  3 – agree  2 - disagree | SD  Disagree  Neither  Agree  SA  Total | 3  14  21  15 (27.8%)  1 ( 1.9%)  54 |

*Conducting rapid evidence synthesis*

- - Knowledge: majority neither agree or disagree that they have high level of knowledge
  - Experience: majority in Georgia neither agrees or disagrees; majority in India, Malaysia and Zimbabwe disagree they have experience
  - Confidence: majority neither agree or disagree that they are confident, except India disagrees they are confident

| **Question** | **Georgia** | **India** | **Malaysia** | **Zimbabwe** | **All** |  |
| --- | --- | --- | --- | --- | --- | --- |
| I currently have a high level of knowledge on how to conduct rapid evidence synthesis and/or rapid reviews. | 5 - neither a or d  5 – disagree  3 – agree  1 – strongly disagree | 1 – neither a or d | 16 – neither a or d  7 – disagree  5 – agree  3 – SD  1 – SA | 4 - neither a or d  3 - disagree  3 - agree  1 – strongly disagree | SD  Disagree  Neither  Agree  SA  Total | 5  15  26  11 (19.0%)  1 ( 1.7%)  58 |
| I have experience conducting rapid evidence synthesis. | 6 - neither a or d  4 – disagree  4 - agree | 1 – disagree | 18 – disagree  4 – neither a or d  4 – agree  6 – SD | 5 - disagree  3 - neither a or d  2 – agree  1 – strongly disagree | SD  Disagree  Neither  Agree  SA  Total | 7  28  13  10 (17.2%)  0  58 |
| I am confident in my ability to conduct rapid evidence synthesis. | 8 - neither a or d  4 – disagree  2 - agree | 1 – disagree | 13 – neither a or d  9 – disagree  8 – agree  2 – SD | 6 - neither a or d  3 – agree  2 - disagree | SD  Disagree  Neither  Agree  SA  Total | 2  16  27  13 (22.4%)  0  58 |

**Q4 - Have you completed any rapid evidence synthesis products to date, (e.g. rapid reviews, rapid policy briefs, rapid evidence summaries, rapid inventories, etc.)?**

| **Georgia** | **India** | **Malaysia** | **Zimbabwe** |
| --- | --- | --- | --- |
| - 10 have completed rapid evidence synthesis product (1 hour-3 month timelines) - Rapid policy brief, evidence brief - Demand and need for this - Engaged patients, researchers, policy makers, service providers | - 1 person said no | - 7 people said yes; 25 said no (4 weeks tp 6 month timelines) - Related to health economics, rapid response and inventory, rapid appraisal - Engaged policy makers/users, MoH - Challenges: lack of experience, literature search, clarifying question/stakeholder engagement - Strengths: leadership, collaborators | - 2 people said they have completed a rapid evidence synthesis product - 1 was not sure what the evidence synthesis product was. |

**Q5. What challenges (barriers) could you/your organization face when conducting rapid evidence synthesis and developing rapid products?**

- Resistance to change
- Buy-in from ministry/policy makers
- Lack of demand
- Lack of experience/knowledge of rapid evidence product methods
- Lack of quality data
- Lack of time/staff/specialists (i.e.,. experts, librarian)
- Unclear questions
- Poor internet connection/access to databases

**Q6. What strengths (facilitators) could you/your organization leverage/utilize to help in conducting rapid evidence synthesis?**

- Leverage people with knowledge/experience in policy analysis and systematic review processes
- Motivated staff
- Team work
- Buy-in for evidence in policy making
- Champions in senior leadership roles
- Strong partnerships/engagement with ministry
- Access to database/sources of information
- Dedicated unit for evidence synthesis

**Q7 - Please rank the topics below in order of importance (i.e., skills needed immediately and/or the team has low knowledge on the topic) by dragging and dropping them. Place the most important topic (i.e., the topic you think your organization needs training on the most) at the top of the list and the least important topic at the bottom of the list.**

|  | = Topic most platforms want, but it is not yet included explicitly on the schedule |
| --- | --- |
|  | = Topic in tentative schedule but not part of ranking topics |
|  | = Topic that only 1 platform wants that is also on schedule |

| **#** | **Georgia** | **India** | **Malaysia** | **Zimbabwe** | **Tentative schedule** |
| --- | --- | --- | --- | --- | --- |
| 1 | Rapid review methods | Engaging policy-makers and health systems managers in the conduct of rapid reviews and development of rapid products | Overview of evidence synthesis methods | Overview of evidence synthesis methods | Overview of evidence synthesis methods |
| 2 | The need for rapid reviews and rapid evidence products to inform health policy and systems | Selecting rapid review methods for complex questions related to health policy and system improvements | The need for rapid reviews and rapid evidence products to inform health policy and systems | The need for rapid reviews and rapid evidence products to inform health policy and systems | Engaging policy-makers and health systems managers in the conduct of rapid reviews and development of rapid products |
| 3 | Systematic review methods | Rapid review methods | Systematic review methods | How to develop rapid evidence synthesis products | Developing a protocol for a rapid review |
| 4 | Overview of evidence synthesis methods | How to develop rapid evidence synthesis products | Rapid review methods | Systematic review methods | How to develop rapid evidence synthesis products |
| 5 | Learning from the experiences of other countries that have successfully implemented similar initiatives | Improving the uptake of rapid evidence synthesis products | How to develop rapid evidence synthesis products | Measuring impact of the ERA platform on policy and decision-making/monitoring and evaluation | Selecting rapid review methods for complex questions related to health policy and system improvements |
| 6 | How to improve quality and efficiency in selecting, abstracting, and appraising studies for rapid reviews | Reporting and disseminating rapid review findings | Engaging policy-makers and health systems managers in the conduct of rapid reviews and development of rapid products | Rapid review methods | How to improve quality and efficiency in selecting, abstracting, and appraising studies for rapid reviews |
| 7 | Fostering the use of rapid evidence synthesis products in health policy in low- and middle- income countries | Stimulating and sustaining demand from policymakers | Stimulating and sustaining demand from policymakers | Engaging policy-makers and health systems managers in the conduct of rapid reviews and development of rapid products | Measuring impact of the ERA platform on policy and decision-making/monitoring and evaluation |
| 8 | Improving the uptake of rapid evidence synthesis products | Measuring impact of the ERA platform on policy and decision-making/monitoring and evaluation | How to conduct rapid reviews in LMICs | Improving the uptake of rapid evidence synthesis products | Learning from the experiences of other countries that have successfully implemented similar initiatives |
| 9 | Measuring impact of the ERA platform on policy and decision-making/monitoring and evaluation | The need for rapid reviews and rapid evidence products to inform health policy and systems | Fostering the use of rapid evidence synthesis products in health policy in low- and middle- income countries | Fostering the use of rapid evidence synthesis products in health policy in low- and middle- income countries | Stimulating and sustaining demand from policymakers |
| 10 | Reporting and disseminating rapid review findings | Learning from the experiences of other countries that have successfully implemented similar initiatives | Selecting rapid review methods for complex questions related to health policy and system improvements | Reporting and disseminating rapid review findings | Reporting and disseminating rapid review findings |

**Q8 - Are there any additional skills or topics you would like to see addressed as a webinar?**

| Monitoring & Evaluation of stakeholder engagement activities like policy dialogues |
| --- |
| Developing the rapid review protocol; Essential list of SOPs; |
| GOALS OF RESEARCH REPORTING RAPID REVIEWS |
| Evidence of the impact of rapid response briefs on the health outcomes in the countries that use and not use evidence-informed health policy |
| rapid review data extraction/synthesis/dissemination |
| Qualitative reviews methodologies |

**Q9 - Are there any additional comments/feedback you would like to provide?**

| Sharing examples/ case studies/ experience (questions asked and the process in the preparation of the response) in training/ webinar will help to reinforce the points/ topics |
| --- |
| Getting good and high quality research evidence (direct, precise, consistent, low RoB and less publication bias), and ability to contextualise the available evidence to produce (any) responses to any requests (even lack of sufficient basis) by policymakers would great challenges to overcome by us mentally and hope to be able to continue in this service as supported by good outcomes arised from it. |

## Appendix A

WHO TAC ERA Program - Learning Needs Assessment - Georgia
Number of survey participants: 14

**Q1 - What is your role in the ERA platform?**

| Project Manager |
| --- |
| Evidence informed health policy and systems decision-making:the role of evidence synthesis |
| Researcher |
| Lead Researcher |
| Identification, analysis and solution of the problem |
| participation and experience in the field |
| My role in the ERA platform is to rise level of knowledge about evidence synthesis, level of knowledge on how to synthesize health policy and systems evidence and level how to conduct systems evidence |

**Q2 - Please rate your level of agreement with each statement on a scale from strongly disagree to strongly agree.**

| # | Field | Minimum | Maximum | Mean | Std Deviation | Variance | Count |
| --- | --- | --- | --- | --- | --- | --- | --- |
| 1 | I currently have a high level of knowledge about evidence synthesis | 2.00 | 4.00 | 3.21 | 0.67 | 0.45 | 14 |
| 2 | I have experience synthesizing evidence | 2.00 | 4.00 | 3.36 | 0.72 | 0.52 | 14 |
| 3 | I am confident in my ability to synthesize evidence | 2.00 | 4.00 | 3.21 | 0.67 | 0.45 | 14 |
| 4 | I currently have a high level of knowledge on how to synthesize health policy and systems evidence | 2.00 | 4.00 | 3.00 | 0.65 | 0.43 | 14 |
| 5 | I have experience synthesizing health policy and systems evidence | 2.00 | 4.00 | 3.14 | 0.74 | 0.55 | 14 |
| 6 | I am confident in my ability to synthesize health policy and systems evidence | 2.00 | 4.00 | 3.14 | 0.64 | 0.41 | 14 |
| 7 | I currently have a high level of knowledge on how to conduct systematic reviews | 1.00 | 4.00 | 2.54 | 0.75 | 0.56 | 13 |
| 8 | I have experience conducting systematic reviews | 1.00 | 4.00 | 2.54 | 0.93 | 0.86 | 13 |
| 9 | I am confident in my ability to conduct a systematic review | 1.00 | 4.00 | 2.58 | 0.95 | 0.91 | 12 |


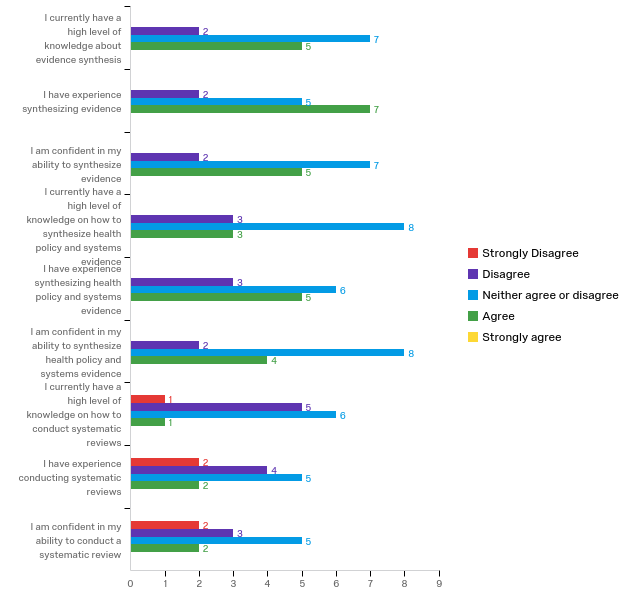


**Q3 - Please rate your level of agreement with each statement on a scale from strongly disagree to strongly agree.**


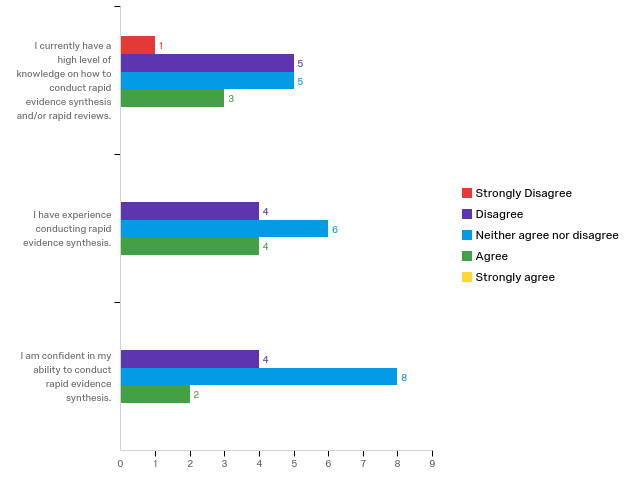


| # | Field | Minimum | Maximum | Mean | Std Deviation | Variance | Count |
| --- | --- | --- | --- | --- | --- | --- | --- |
| 1 | I currently have a high level of knowledge on how to conduct rapid evidence synthesis and/or rapid reviews. | 1.00 | 4.00 | 2.71 | 0.88 | 0.78 | 14 |
| 2 | I have experience conducting rapid evidence synthesis. | 2.00 | 4.00 | 3.00 | 0.76 | 0.57 | 14 |
| 3 | I am confident in my ability to conduct rapid evidence synthesis. | 2.00 | 4.00 | 2.86 | 0.64 | 0.41 | 14 |

**Q4 - Have you completed any rapid evidence synthesis products to date, (e.g. rapid reviews, rapid policy briefs, rapid evidence summaries, rapid inventories, etc.)?**


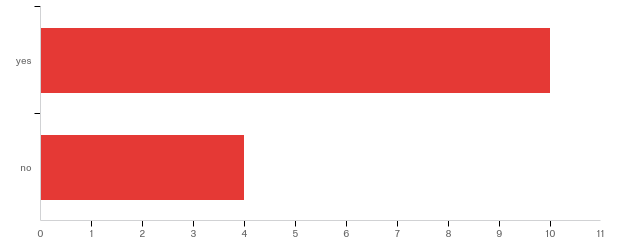


| # | Answer | % | Count |
| --- | --- | --- | --- |
| 1 | yes | 71.43% | 10 |
| 2 | no | 28.57% | 4 |
|  | Total | 100% | 14 |

**Q4a - What rapid review products did you complete?**

| None |
| --- |
| Rapid policy brief and evidence briefs |
| none |
| Review of the draft law |
| Policy brief, information papers, fanctually papers and etc. |
| Policy breif |

**Q4c - Who were the stakeholders involved?**

| Patients, Service providers, Researchers, Policy Makers |
| --- |
| field experts, no policy makers were involved |
| Competent departments and specialists |
| Service providers, vulnerable groups |
| No one |

**Q4d - What was your approach to engaging these stakeholders?**

| Workshops, face to face meetings |
| --- |
| we involved field experts with whom we worked during that specific activity, based on which rapid product was developed |
| Positive |
| Obtain information |

**Q4e - What stage of the review cycle did you engage these stakeholders in? (Please note any challenges and successes experienced when working with stakeholders on rapid reviews).**

| In the initial and/or final phases mostly. |
| --- |
| end stage of the rapid product development |
| Almost every stage |
| 1 |

**Q4f - Within what time frame were the rapid products completed?**

| I complete policy briefs within 2 weeks-1 month |
| --- |
| Up two 3 months |
| 1.5 - 2 months |
| From one week to six months |
| depends product tipe. from hour to 10 days. |
| 1 |

**Q4g - What challenges (barriers) did you/your organization encounter when developing the rapid products?**

| Deadlines are tight and it is somehow tricky to ensure stakeholders engagement in the process on time. |
| --- |
| no extensive practice of rapid products development, no formal training with this regards |
| Social welfare and risk consideration |
| There is not enough information resources |

**Q4h - What strengths (facilitators) did you/your organization leverage to develop the rapid products?**

| The organization has good understanding of how health systems work and this knowledge is a added value to develop quality product. The second factor is that organization legal status (NGO) gives the possibility to avoid some bureaucratic procedures and time saved consume on products preparation. |
| --- |
| access to evidences, search skills, knowledge synthesis skills, health systems knowledge |
| Demand and need |
| human resource |

**Q5 - What challenges (barriers) could you/your organization face when conducting rapid evidence synthesis and developing rapid products?**

| Lack of experience, low engagement from policy makers |
| --- |
| Better analysis of legislation Before, during and after its passage through Parliament More research in support of oversight/scrutiny role |

**Q6 - What strengths (facilitators) could you/your organization leverage/utilize to help in conducting rapid evidence synthesis and developing rapid products?**

| Strong partnership relationships with parliament, MoH and NCD. Solid reputation of our organization. |
| --- |
| Impartial – working for MPs of all parties Subject expertise A bridge to the outside world,Pro-active and responsive Support to plenary, committees and individual MPs |

**Q7 - Please rank the topics below in order of importance (i.e., skills needed immediately and/or the team has low knowledge on the topic) by dragging and dropping them. Place the most important topic (i.e., the topic you think your organization needs training on the most) at the top of the list and the least important topic at the bottom of the list.**

| # | Question |
| --- | --- |
| 1 | Rapid review methods |
| 2 | The need for rapid reviews and rapid evidence products to inform health policy and systems |
| 3 | Systematic review methods |
| 4 | Overview of evidence synthesis methods |
| 5 | Learning from the experiences of other countries that have successfully implemented similar initiatives |
| 6 | How to improve quality and efficiency in selecting, abstracting, and appraising studies for rapid reviews |
| 7 | Fostering the use of rapid evidence synthesis products in health policy in low- and middle- income countries |
| 8 | Improving the uptake of rapid evidence synthesis products |
| 9 | Measuring impact of the ERA platform on policy and decision-making/monitoring and evaluation |
| 10 | Reporting and disseminating rapid review findings |

**Q8 - Are there any additional skills or topics you would like to see addressed as a webinar?**

| GOALS OF RESEARCH REPORTING RAPID REVIEWS |
| --- |

**Q9 - Are there any additional comments/feedback you would like to provide?**

| Able to access external sources to obtain the information needed |
| --- |

### WHO TAC ERA Program - Learning Needs Assessment – India

Number of survey participants: 1

**Q1 - What is your role in the ERA platform?**

| *Removed for confidentiality reasons.* |
| --- |

**Q2 - Please rate your level of agreement with each statement on a scale from strongly disagree to strongly agree.**

| # | Field | Minimum | Maximum | Mean | Std Deviation | Variance | Count |
| --- | --- | --- | --- | --- | --- | --- | --- |
| 1 | I currently have a high level of knowledge about evidence synthesis | 3.00 | 3.00 | 3.00 | 0.00 | 0.00 | 1 |
| 2 | I have experience synthesizing evidence | 4.00 | 4.00 | 4.00 | 0.00 | 0.00 | 1 |
| 3 | I am confident in my ability to synthesize evidence | 3.00 | 3.00 | 3.00 | 0.00 | 0.00 | 1 |
| 4 | I currently have a high level of knowledge on how to synthesize health policy and systems evidence | 4.00 | 4.00 | 4.00 | 0.00 | 0.00 | 1 |
| 5 | I have experience synthesizing health policy and systems evidence | 4.00 | 4.00 | 4.00 | 0.00 | 0.00 | 1 |
| 6 | I am confident in my ability to synthesize health policy and systems evidence | 4.00 | 4.00 | 4.00 | 0.00 | 0.00 | 1 |
| 7 | I currently have a high level of knowledge on how to conduct systematic reviews | 4.00 | 4.00 | 4.00 | 0.00 | 0.00 | 1 |
| 8 | I have experience conducting systematic reviews | 4.00 | 4.00 | 4.00 | 0.00 | 0.00 | 1 |
| 9 | I am confident in my ability to conduct a systematic review | 4.00 | 4.00 | 4.00 | 0.00 | 0.00 | 1 |


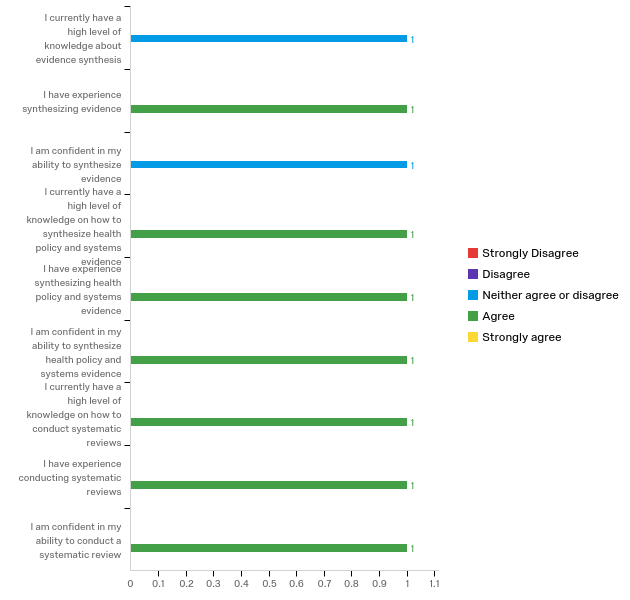


**Q3 - Please rate your level of agreement with each statement on a scale from strongly disagree to strongly agree.**


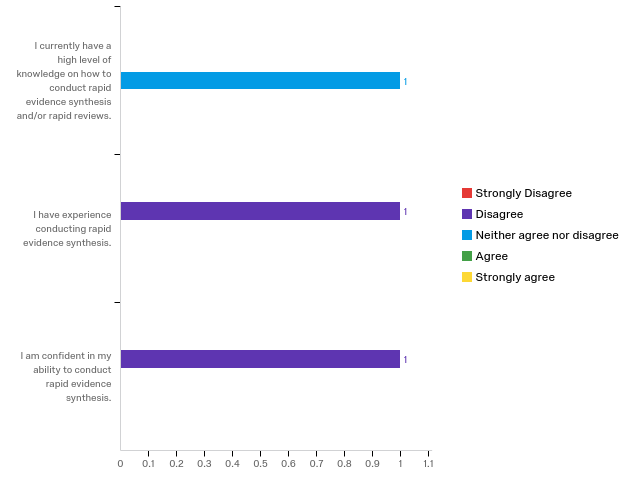


| # | Field | Minimum | Maximum | Mean | Std Deviation | Variance | Count |
| --- | --- | --- | --- | --- | --- | --- | --- |
| 1 | I currently have a high level of knowledge on how to conduct rapid evidence synthesis and/or rapid reviews. | 3.00 | 3.00 | 3.00 | 0.00 | 0.00 | 1 |
| 2 | I have experience conducting rapid evidence synthesis. | 2.00 | 2.00 | 2.00 | 0.00 | 0.00 | 1 |
| 3 | I am confident in my ability to conduct rapid evidence synthesis. | 2.00 | 2.00 | 2.00 | 0.00 | 0.00 | 1 |

**Q4 - Have you completed any rapid evidence synthesis products to date, (e.g. rapid reviews, rapid policy briefs, rapid evidence summaries, rapid inventories, etc.)?**


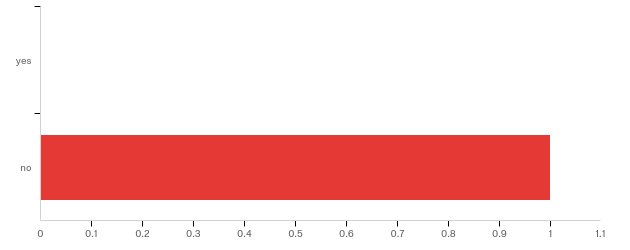


| # | Field | Minimum | Maximum | Mean | Std Deviation | Variance | Count |
| --- | --- | --- | --- | --- | --- | --- | --- |
| 1 | Have you completed any rapid evidence synthesis products to date, (e.g. rapid reviews, rapid policy briefs, rapid evidence summaries, rapid inventories, etc.)? | 2.00 | 2.00 | 2.00 | 0.00 | 0.00 | 1 |

**Q5 - What challenges (barriers) could you/your organization face when conducting rapid evidence synthesis and developing rapid products?**

| questions are not clear, timelines are especially tight, and also there is very little evidence of good quality to make a recommendation, which then lowers expectations and demands from policymakers |
| --- |

**Q6 - What strengths (facilitators) could you/your organization leverage/utilize to help in conducting rapid evidence synthesis and developing rapid products?**

| we have a unit working specifically on evidence synthesis and are positioning ourselves in a leadership role in India in this space. We are also already working wiith various government departments in this space and have a number of state level HPSR projects in place that lend themselves to extending and integrating RES at the state level (in India, health is a state subject). |
| --- |

**Q7 - Please rank the topics below in order of importance (i.e., skills needed immediately and/or the team has low knowledge on the topic) by dragging and dropping them. Place the most important topic (i.e., the topic you think your organization needs training on the most) at the top of the list and the least important topic at the bottom of the list.**

| # | Question |
| --- | --- |
| 1 | Engaging policy-makers and health systems managers in the conduct of rapid reviews and development of rapid products |
| 2 | Selecting rapid review methods for complex questions related to health policy and system improvements |
| 3 | Rapid review methods |
| 4 | How to develop rapid evidence synthesis products |
| 5 | Improving the uptake of rapid evidence synthesis products |
| 6 | Reporting and disseminating rapid review findings |
| 7 | Stimulating and sustaining demand from policymakers |
| 8 | Measuring impact of the ERA platform on policy and decision-making/monitoring and evaluation |
| 9 | The need for rapid reviews and rapid evidence products to inform health policy and systems |
| 10 | Learning from the experiences of other countries that have successfully implemented similar initiatives |

**Q9 - Are there any additional comments/feedback you would like to provide?**

| We have to bear in mind that national elections are underway in India. Some guidance on political economy and broader contextual factors and how they affect ERA platforms will be very useful. |
| --- |

WHO TAC ERA Program - Learning Needs Assessment - Malaysia
Number of survey participants: 32

**Q1 - What is your role in the ERA platform?**

| team leader |
| --- |
| researcher |
| researcher |
| researcher |
| conduct rapid review |
| advisor |
| Team member - Human Resources for Health |
| Team Member - Human Resources for Health |
| Stakehder representative, database development |
| Researcher |
| Research assistant |
| Rapid review |
| Policy-maker representative |
| Participant |
| Department Champion/ Reviewer |
| Contribute to search strategy, data/evidence interpretation, academic content and scientific writing of the rapid response briefs. |
| Contribute to developing and reviewing as well as training and |
| Contribute to developing and reviewer of rapid reviews, training |
| Conduct rapid reviews |
| Conduct rapid review, |
| Conduct Rapid Review |
| Conduct Rapid Review |
| Conduct Rapid Review |
| Co-leader |
| Co principal investigator / reseracher |

**Q2 - Please rate your level of agreement with each statement on a scale from strongly disagree to strongly agree.**

| # | Field | Minimum | Maximum | Mean | Std Deviation | Variance | Count |
| --- | --- | --- | --- | --- | --- | --- | --- |
| 1 | I currently have a high level of knowledge about evidence synthesis | 1.00 | 5.00 | 3.22 | 0.86 | 0.73 | 32 |
| 2 | I have experience synthesizing evidence | 2.00 | 5.00 | 3.31 | 0.92 | 0.84 | 32 |
| 3 | I am confident in my ability to synthesize evidence | 2.00 | 5.00 | 3.31 | 0.81 | 0.65 | 32 |
| 4 | I currently have a high level of knowledge on how to synthesize health policy and systems evidence | 1.00 | 4.00 | 2.69 | 0.92 | 0.84 | 32 |
| 5 | I have experience synthesizing health policy and systems evidence | 1.00 | 5.00 | 2.66 | 0.96 | 0.91 | 32 |
| 6 | I am confident in my ability to synthesize health policy and systems evidence | 1.00 | 4.00 | 2.91 | 0.84 | 0.71 | 32 |
| 7 | I currently have a high level of knowledge on how to conduct systematic reviews | 1.00 | 5.00 | 2.94 | 0.90 | 0.81 | 32 |
| 8 | I have experience conducting systematic reviews | 1.00 | 5.00 | 2.91 | 1.01 | 1.02 | 32 |
| 9 | I am confident in my ability to conduct a systematic review | 1.00 | 5.00 | 3.06 | 0.93 | 0.87 | 32 |


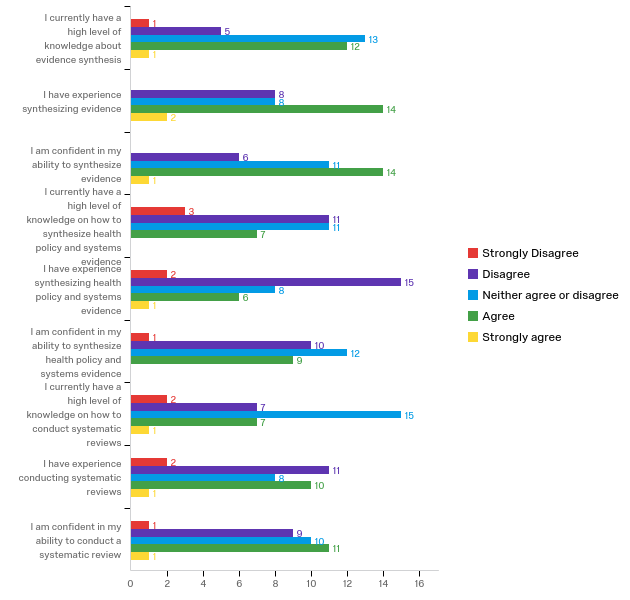


**Q3 - Please rate your level of agreement with each statement on a scale from strongly disagree to strongly agree.**


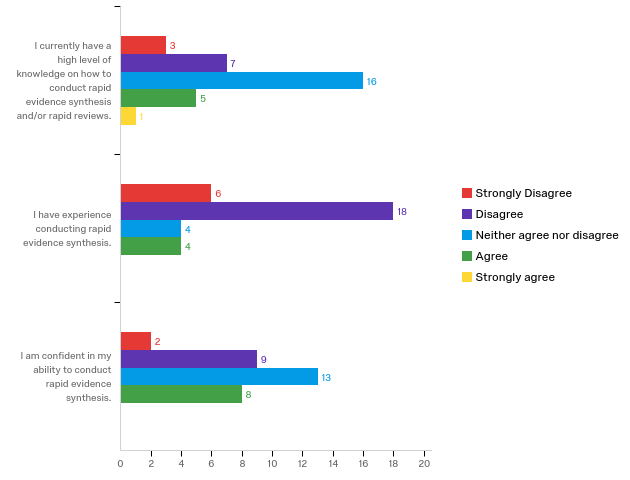


| # | Field | Minimum | Maximum | Mean | Std Deviation | Variance | Count |
| --- | --- | --- | --- | --- | --- | --- | --- |
| 1 | I currently have a high level of knowledge on how to conduct rapid evidence synthesis and/or rapid reviews. | 1.00 | 5.00 | 2.81 | 0.92 | 0.84 | 32 |
| 2 | I have experience conducting rapid evidence synthesis. | 1.00 | 4.00 | 2.19 | 0.88 | 0.78 | 32 |
| 3 | I am confident in my ability to conduct rapid evidence synthesis. | 1.00 | 4.00 | 2.84 | 0.87 | 0.76 | 32 |

**Q4 - Have you completed any rapid evidence synthesis products to date, (e.g. rapid reviews, rapid policy briefs, rapid evidence summaries, rapid inventories, etc.)?**


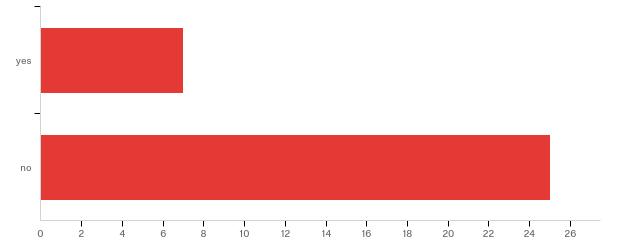


| # | Answer | % | Count |
| --- | --- | --- | --- |
| 1 | yes | 21.88% | 7 |
| 2 | no | 78.13% | 25 |
|  | Total | 100% | 32 |

**Q4a - What rapid review products did you complete?**

| Technology Report |
| --- |
| In process |
| Mostly related to Health Economics Area |
| rapid response & inventory |
| Rapid Appraisal |

**Q4b - How many rapid products have you completed?**

| 1 |
| --- |
| In process |
| One |
| two |
| 1 |

**Q4c - Who were the stakeholders involved?**

| Policy makers, users |
| --- |
| Very minimal |
| MoH Medical development division |
| Medical Planning Division |

**Q4d - What was your approach to engaging these stakeholders?**

| Discussion |
| --- |
| Frequent meeting and presentation of the findings |
| interative |
| Meeting and Stakeholder Dialogue |

**Q4e - What stage of the review cycle did you engage these stakeholders in? (Please note any challenges and successes experienced when working with stakeholders on rapid reviews).**

| In Process |
| --- |
| throughout |
| Conceptualize & Design as well as Uptake & Evaluation |

**Q4f - Within what time frame were the rapid products completed?**

| 4 weeks |
| --- |
| Not sure |
| Two months |
| less than 1 year |
| About 3-6 months |

**Q4g - What challenges (barriers) did you/your organization encounter when developing the rapid products?**

| Searching evidence/articles |
| --- |
| In Process |
| Lack of experience in conducting the review |
| article and literature search and retrieval |
| Clarifying the question, engaging the relevant stakeholders |

**Q4h - What strengths (facilitators) did you/your organization leverage to develop the rapid products?**

| Good leader |
| --- |
| In Process |
| Systematic searching and summarizing the evidence |
| collaborators |
| Under the same ministry, dedicated team members & good rapport with stakeholder |

**Q5 - What challenges (barriers) could you/your organization face when conducting rapid evidence synthesis and developing rapid products?**

| Don' t have enough knowledge or skills in conducting rapid evidence synthesis and developing rapid products. |
| --- |
| Establish good rapport with policy maker and making awareness on availablity of this service |
| Problems in assessing full article in database |
| Clarifying questions with stakeholders, unavailability of an information specialist/librarian |
| manpower, produce within timely manner as requested by stakeholders |
| clear question and data to support question |
| lack of sharing products |
| access to journals |
| lack of expert |
| time |
| adequate knowledge and dedicated time to conduct the rapid review |
| Skills, knowledge and resources |
| To set aside time to focus on RR since members are involved in other commitments |
| Lack of exposure |
| Lack of information specialist, access to database of references, data abstractions skills |
| Protected time to do the review |
| Time available/ given (not the core job), trained staff, good availability of information/ data, formulating the question (not clear/ vague/ too wide), knowledge in good report writing |
| Translating data into evidence in a timely manner that truly represents the local population |
| Literature search |
| lack of knowledge |
| Interface with the policy makers-eliciting a question from policy makers that is worth synthesizing rapid evidence for, |
| Personally, it is about interest in the topic given or requested by the policymakers to conduct the rapid review. This will related to having sufficient or good motivation to complete the rapid review or rapid response brief. The other more general barriers that I foresee are obtaining adequate cooperation from key informants, input and trust from local experts, access to the relevant local data/evidence and good quality research evidence to produce and contextualise the rapid response briefs. |
| time |

**Q6 - What strengths (facilitators) could you/your organization leverage/utilize to help in conducting rapid evidence synthesis and developing rapid products?**

| Samples of completed Rapid review from other countries and employing research assistants |
| --- |
| Easily accessed full article paper |
| Roping in experts from the academic side with experience in systematic reviews to help facilitate in designing the research question with the stakeholders and the first few steps in the conduct of evidence synthesis |
| strong engagement with stakeholders |
| network available |
| know how plan, conduct & review |
| quality journal articles |
| enough man-power |
| team work |
| good teamwork and dedication among our rapid review team members |
| Content expertise, training and experience in systematic reviews |
| To have academician highly skilled in conducting systematic review to be in the team |
| Do awareness and training among staff |
| Subject matter expert |
| Ready demand/ needs for the service, good management support, staff with some knowledge of evidence synthesis and assessment |
| Access to database; experience in conducting systematic review |
| Expert team members |
| good team work |
| To coordinate the involvement of experts from various institutions eg MOH, universities and private centres to conduct an efficient RR |
| Commitment of the rapid response brief centre or coordinator (Institute of Health System Research Malaysia); experiences and knowledge of systematic reviews, knowledge in clinical epidemiology, scientific research and academic writing. |
| experience in evidence synthesis |

**Q7 - Please rank the topics below in order of importance (i.e., skills needed immediately and/or the team has low knowledge on the topic) by dragging and dropping them. Place the most important topic (i.e., the topic you think your organization needs training on the most) at the top of the list and the least important topic at the bottom of the list.**

| # | Question |
| --- | --- |
| 1 | Overview of evidence synthesis methods |
| 2 | The need for rapid reviews and rapid evidence products to inform health policy and systems |
| 3 | Systematic review methods |
| 4 | Rapid review methods |
| 5 | How to develop rapid evidence synthesis products |
| 6 | Engaging policy-makers and health systems managers in the conduct of rapid reviews and development of rapid products |
| 7 | Stimulating and sustaining demand from policymakers |
| 8 | How to conduct rapid reviews in LMICs |
| 9 | Fostering the use of rapid evidence synthesis products in health policy in low- and middle- income countries |
| 10 | Selecting rapid review methods for complex questions related to health policy and system improvements |

**Q8 - Are there any additional skills or topics you would like to see addressed as a webinar?**

| rapid review data extraction/synthesis/dissemination |
| --- |
| Qualitative reviews methodologies |
| - |
| 1. In Overview: Difference and similarities of the different methods and products/reports (systematic reviews, policy brief, rapid response) 2. Documentation (guidelines, database of reports [for easy retrieval]) etc. |
| Evidence of the impact of rapid response briefs on the health outcomes in the countries that use and not use evidence-informed health policy. This may provides continuing encouragement to improve and expand this effort. |

**Q9 - Are there any additional comments/feedback you would like to provide?**

| Very good and beneficial workshop |
| --- |
| Sharing examples/ case studies/ experience (questions asked and the process in the preparation of the response) in training/ webinar will help to reinforce the points/ topics |
| Getting good and high quality research evidence (direct, precise, consistent, low RoB and less publication bias), and ability to contextualise the available evidence to produce (any) responses to any requests (even lack of sufficient basis) by policymakers would great challenges to overcome by us mentally and hope to be able to continue in this service as supported by good outcomes arised from it. |

### WHO TAC ERA Program - Learning Needs Assessment - Zimbabwe

Number of survey participants: 11

**Q1 - What is your role in the ERA platform?**

| technical reviewer |
| --- |
| part of the core team |
| Technical external Ministry of Health Partner |
| Policy research and analysis |
| I provide technical support to the Ministry of Health and Child Care ERAZ team |
| Student |
| Technical Support |
| Participant |
| Project Lead |
| Librarian |

**Q2 - Please rate your level of agreement with each statement on a scale from strongly disagree to strongly agree.**

| # | Field | Minimum | Maximum | Mean | Std Deviation | Variance | Count |
| --- | --- | --- | --- | --- | --- | --- | --- |
| 1 | I currently have a high level of knowledge about evidence synthesis | 2.00 | 4.00 | 2.82 | 0.72 | 0.51 | 11 |
| 2 | I have experience synthesizing evidence | 2.00 | 4.00 | 2.91 | 0.90 | 0.81 | 11 |
| 3 | I am confident in my ability to synthesize evidence | 2.00 | 4.00 | 3.00 | 0.74 | 0.55 | 11 |
| 4 | I currently have a high level of knowledge on how to synthesize health policy and systems evidence | 2.00 | 4.00 | 2.73 | 0.62 | 0.38 | 11 |
| 5 | I have experience synthesizing health policy and systems evidence | 1.00 | 4.00 | 2.45 | 0.78 | 0.61 | 11 |
| 6 | I am confident in my ability to synthesize health policy and systems evidence | 2.00 | 4.00 | 3.10 | 0.70 | 0.49 | 10 |
| 7 | I currently have a high level of knowledge on how to conduct systematic reviews | 2.00 | 4.00 | 3.10 | 0.54 | 0.29 | 10 |
| 8 | I have experience conducting systematic reviews | 2.00 | 4.00 | 2.80 | 0.75 | 0.56 | 10 |
| 9 | I am confident in my ability to conduct a systematic review | 2.00 | 4.00 | 3.09 | 0.67 | 0.45 | 11 |


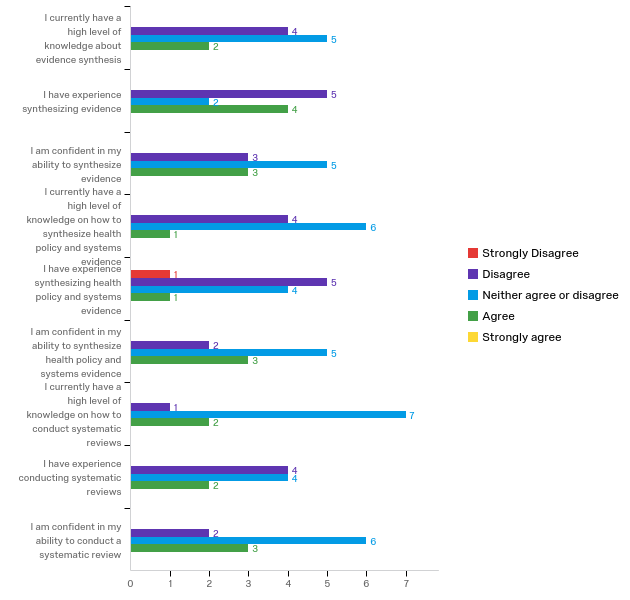


**Q3 - Please rate your level of agreement with each statement on a scale from strongly disagree to strongly agree.**


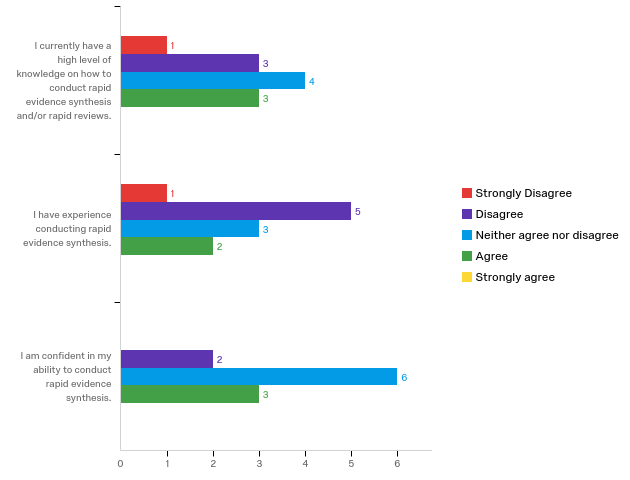


| # | Field | Minimum | Maximum | Mean | Std Deviation | Variance | Count |
| --- | --- | --- | --- | --- | --- | --- | --- |
| 1 | I currently have a high level of knowledge on how to conduct rapid evidence synthesis and/or rapid reviews. | 1.00 | 4.00 | 2.82 | 0.94 | 0.88 | 11 |
| 2 | I have experience conducting rapid evidence synthesis. | 1.00 | 4.00 | 2.55 | 0.89 | 0.79 | 11 |
| 3 | I am confident in my ability to conduct rapid evidence synthesis. | 2.00 | 4.00 | 3.09 | 0.67 | 0.45 | 11 |

**Q4 - Have you completed any rapid evidence synthesis products to date, (e.g. rapid reviews, rapid policy briefs, rapid evidence summaries, rapid inventories, etc.)?**


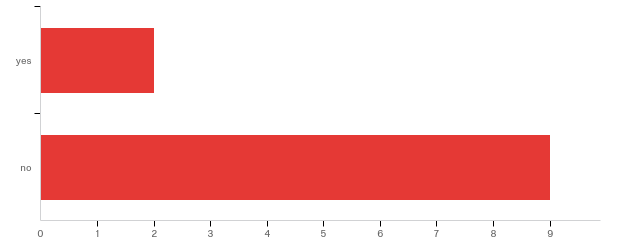


| # | Answer | % | Count |
| --- | --- | --- | --- |
| 1 | yes | 18.18% | 2 |
| 2 | no | 81.82% | 9 |
|  | Total | 100% | 11 |

**Q4a - What rapid review products did you complete?**

| am not sure |
| --- |

**Q4f - Within what time frame were the rapid products completed?**

| two weeks |
| --- |

**Q5 - What challenges (barriers) could you/your organization face when conducting rapid evidence synthesis and developing rapid products?**

| Resistance to change from those who have been in the system for too long |
| --- |
| Buy in at all levels in the Ministry is key |
| Limited internet connection, no hands on experience in conducting rapid evidence synthesis |
| Quality and reliable data |
| Limited access to high level policymakers, Limited access to peer reviewed journals and databases, Limited evidence synthesis skills, Lack of motivation and incentives, Government Bureaucracy |
| knowledge gaps |
| Lack of time; Not enough experienced mentors |
| Lack of knowledge and skills |

**Q6 - What strengths (facilitators) could you/your organization leverage/utilize to help in conducting rapid evidence synthesis and developing rapid products?**

| the organisation has officials who have strength in p[policy analysis and can be utilized in the project |
| --- |
| Basic understanding of the systematic review processes which is leveraged upon to produce rapid evidence sysnthesis |
| ready to learn human resources and a well resourced library |
| gathering and analysing evidence |
| Awareness of the importance of using evidence in policymaking, High level buy-in, Capacity development & Support from AHPSR, Provisions in policy and other strategic documents like National Health Strategy, Existing institutional Networks, Well educated staff and allied existing roles |
| experts in different areas |
| Existence of this funded ERA opportunity; Availability of ZEIPNET partners to mentor us; There are many rapid products that come out of thePolicy & Planning Department, where the ERA is now housed; We have a keen and vibrant champion among th high-level leadership. |

**Q7 - Please rank the topics below in order of importance (i.e., skills needed immediately and/or the team has low knowledge on the topic) by dragging and dropping them. Place the most important topic (i.e., the topic you think your organization needs training on the most) at the top of the list and the least important topic at the bottom of the list.**

| # | Question |
| --- | --- |
| 1 | Overview of evidence synthesis methods |
| 2 | The need for rapid reviews and rapid evidence products to inform health policy and systems |
| 3 | How to develop rapid evidence synthesis products |
| 4 | Systematic review methods |
| 5 | Measuring impact of the ERA platform on policy and decision-making/monitoring and evaluation |
| 6 | Rapid review methods |
| 7 | Engaging policy-makers and health systems managers in the conduct of rapid reviews and development of rapid products |
| 8 | Improving the uptake of rapid evidence synthesis products |
| 9 | Fostering the use of rapid evidence synthesis products in health policy in low- and middle- income countries |
| 10 | Reporting and disseminating rapid review findings |

**Q8 - Are there any additional skills or topics you would like to see addressed as a webinar?**

| Monitoring & Evaluation of stakeholder engagement activities like policy dialogues |
| --- |
| Developing the rapid review protocol; Essential list of SOPs; |

**Q9 - Are there any additional comments/feedback you would like to provide?**

| I think this is a good initiative as evidence based decision tend to save on the much scarce health resources |
| --- |
| Knowledge on the subject area is still limited |
| Please share samples of a rapid review protocol, report, rapid products; SOPs |


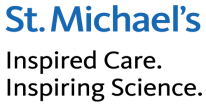

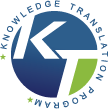


# Appendix 4: Webinar Evaluation Form

**Date of webinar: [Add for each webinar]**

Location of ERA platform:

Role in ERA Platform:

Please indicate how you participated in the WHO TAC ERA webinar:

Attended with group (live)

Attended with group (archived)

Attended webcast individually (live)

Attended webcast individually (archived)

**Webinar title: [Add for each webinar]**

**Learning objectives:**

1. **Add for each session**
2. **Add for each session**
3. **Add for each session**

**Strongly Disagree** **Strongly Agree**

1 2 3 4 5

**A. Webinar Presenter (s) Pre-populate for each session**

**Circle appropriate number:**

1. Demonstrated knowledge of topic 1 2 3 4 5

2. Clear and well organized 1 2 3 4 5

3. Balanced and unbiased presentation 1 2 3 4 5

4. Stimulated enthusiasm about topic 1 2 3 4 5

5. Invited questions and audience interaction 1 2 3 4 5

6. Met all session objectives (see page 2) 1 2 3 4 5

**B. Technical Aspects**

1. The webinar was well organized 1 2 3 4 5

2. I could clearly hear the presenter(s) 1 2 3 4 5

3. There was enough time for interaction/ active learning 1 2 3 4 5

4. I would attend other WHO TAC Program webinars 1 2 3 4 5

**C. Overall evaluation of webinar**

**Unsatisfactory Average Outstanding**

1 2 3 4 5

**D. What did you enjoy about this webinar?**

**E. Is there anything that could have been improved on in this webinar?**

**F. What did you learn from this webinar?**

**G. Besides the topics outlined in the syllabus, are there any other topics you would like us to cover in these webinars that would help your ERA platform produce policy-relevant rapid reviews?**

**H. Any additional comments?**

**Thank you for taking time to fill out the evaluation!**

**Please return by email to** [**amogk@smh.ca**](mailto:amogk@smh.ca)

# Appendix 5: Sample In-Country Workshop Agenda

| \|  \| \| \| \| \| \| --- \| --- \| --- \| --- \| --- \| \| **Time** \| **Duration** \| **Session Title** \| **Objectives of the Session** \| **Facilitator(s)** \| \| 8:30 – 9:00 \| 30 min \| Registration \|  \|  \| \| 9:00-9:30 \| 30 min \| Welcome \|  \|  \| \| 9:30-10:00 \| 30 min \| **Session 1**: Opening and introduction \| 1. Participants introduce themselves using a guide of the pre-workshop tasks \|  \| \| 10:00-10:20 \| 20 min \| **Session 2**: Introduction and overview of the technical training session \| 1. To review the workshop format, content and expectations \|  \| \| 10:20-10:50 \| 25min \| Tea/Coffee break \| \| \| \| 10:50-12:00 \| 70 min \| **Session 3**: Overview of health systems and public policy \| 1. To review the basic understanding and elements of health systems 2. To review participants’ understanding of public policy as a phenomenon and as a process \|  \| \| 12:00-13:00 \| 60 min \| **Session 4**: Overview of EIHP \| 1. To recap on the basic understanding and tenets of evidence-informed decision making in public policy \|  \| \| 13:00-14:00 \| 60 min \| Lunch break \| \| \| \| 14:00-15:45 \| 105 min \| **Session 5**: The EIP framework (problem, policy options, implementation strategies) \| 1. To review participants’’ knowledge of what the EIP framework is and what it does 2. To review participants’ understanding of how to clarify policy problems 3. To review participants’ understanding of identifying and describing policy options 4. To review participants’’ understanding of identifying and presenting implementation strategies \|  \| \| 15:45-16:00 \| 15 min \| Tea/Coffee break \| \| \| \| 16:00-16:40 \| 40 min \| **Session 6**: Rapid Response Services – what are they? \| 1. To introduce to participants’ the basics of a rapid response service for health systems policy questions \|  \| \| 16:40-17:00 \| 20 min \| **Session 7**: RRS questions \| 1. To review policy questions that participants will be working on over the next few days within each group (5mins per group) \|  \| \| Wrapping up of Day 1 \| 1. To receive feedback in light of the day and training in general so as to make necessary adjustments \|  \| **16^th^ May 2019** \| \| \| \| \| \| \| \| --- \| --- \| --- \| --- \| --- \| --- \| --- \| \| **Time** \| \| **Duration** \| \| **Session Title** \| **Objectives of the Session** \| **Facilitator(s)** \| \| \| 8:30-8:45 \| 15 min \| \| Outlook on Day 2 \| \|  \|  \| \| 8:45-10:30 \| 105 min \| \| **Session 7**: Rapid Response Services – tools \| \| 1. To introduce to participants’ the tools used to aid the process on a rapid response service for health systems policy questions \|  \| \| 10:30-11:00 \| 30 min \| \|  \| \| \| \| \| 11:00-13:00 \| 120 min \| \| **Session 8**: Rapid Response Services –  Process  Receiving and clarifying a policy question \| \| 1. To introduce to participants’ the tools used to aid the process on a rapid response service for health systems policy questions 2. To go through the process of receiving and clarifying a question on a RRS \|  \| \| 13:00-14:00 \| 60 min \| \| Lunch break \| \| \| \| \| 14:00-15:30 \| 120 min \| \| ***Session 9:** Developing a search strategy \| \| 1. To have an overview of on how to formulate an effective search strategy for evidence to be used in a RR brief \|  \| \| 15:30-15:50 \| 20 min \| \| Tea break \| \| \| \| \| 15:50-17:00 \| 70 min \| \| **Session 10: Practical session** \| \| 1. To enable participants practice clarifying and developing search strategies for their policy questions \| All facilitators \| \| **17^th^ May 2019** \| \| \| \| \| \| \| \| **Time** \| \| **Duration** \| \| **Session Title** \| **Objectives of the Session** \| **Facilitator(s)** \| \| 8:30- 8:45 \| 15 min \| \| Review of day 2  Outlook on day 3 \| \|  \|  \| \| 8:45-10:30 \| 105 min \| \| ***Session 11 (a)**: Finding evidence (searching for local and global evidence) \| \| 1. To get an overview or recap on how and where to search effectively for research evidence to use in a RR brief 2. A practical session for participants to locate evidence for their clarified policy questions \|  \| \| 10:30-11:00 \| 30 min \| \|  \| \| \| \| \| 11:00-13:00 \| 120 min \| \| **Session 11 (b)**: Assessing the evidence \| \| 1. To learn or recap methods to assess the quality of research evidence for use in a RR brief 2. A practical session in which participants assess local and global evidence located for their policy questions \|  \| \| 13:00-14:00 \| 60 min \| \|  \| \| \| \| \| 14:00-15:30 \| 45 min \| \| **Session 12**: Rapid Response services – Summarizing the evidence \| \| 1. To review different methods of summarizing research evidence in a RR brief \|  \| \| 16:00-18:00 \| 120 min \| \| **Session 12: Practical session**  ***(with a working tea/coffee)*** \| \| 1. To enable participants work on their RR briefs, locating evidence and appraising it \| All facilitators \|   **These sessions will only provide an overview as they will be handled more in-depth in a later training.* |
| --- | --- | --- | --- | --- | --- | --- | --- | --- | --- | --- | --- | --- | --- | --- | --- | --- | --- | --- | --- | --- | --- | --- | --- | --- | --- | --- | --- | --- | --- | --- | --- | --- | --- | --- | --- | --- | --- | --- | --- | --- | --- | --- | --- | --- | --- | --- | --- | --- | --- | --- | --- | --- | --- | --- | --- | --- | --- | --- | --- | --- | --- | --- | --- | --- | --- | --- | --- | --- | --- | --- | --- | --- | --- | --- | --- | --- | --- | --- | --- | --- | --- | --- | --- | --- | --- | --- | --- | --- | --- | --- | --- | --- | --- | --- | --- | --- | --- | --- | --- | --- | --- | --- | --- | --- | --- | --- | --- | --- | --- | --- | --- | --- | --- | --- | --- | --- | --- | --- | --- | --- | --- | --- | --- | --- | --- | --- | --- | --- | --- | --- | --- | --- | --- | --- | --- | --- | --- | --- | --- | --- | --- | --- | --- | --- | --- | --- | --- | --- | --- | --- | --- | --- | --- | --- | --- | --- | --- | --- | --- | --- | --- | --- | --- | --- | --- | --- | --- | --- | --- | --- | --- | --- | --- | --- | --- | --- | --- | --- | --- | --- | --- | --- | --- | --- | --- | --- | --- | --- | --- | --- | --- | --- | --- | --- | --- | --- | --- | --- | --- | --- | --- | --- | --- | --- | --- | --- |

# Appendix 6: Sample Consult Call Meeting Agenda

Date: June 13, 2019

Time: 7:00am-8:00am (EST)/7:00pm-8:00pm MYT

Location: Webex

Attendees:

Regrets:

This is the 2^nd^ of 2 consult calls the MAera platform will have with the Toronto TAC ERA support team. This consult call is scheduled after completion of the in-country workshop in an effort to ensure continued support is available as the platform implements the applied training to their setting.

The purpose of this call is to provide tailored technical assistance to the MAera platform as they continue to establish their process and systems of embedding rapid evidence synthesis into health systems decision-making. The content of the call will be driven and informed by members of the MAera platform. It will provide an opportunity for members to discuss their progress and receive additional support from the Toronto TAC ERA team.

## Agenda

1. Key successes of establishing your platform since you began this initiative in summer 2018.
   1. What goals/milestones were set and accomplished?
   2. What unanticipated accomplishments have you experienced?
2. Challenges you have experienced in establishing your platform.
   1. How have you addressed these challenges?
   2. Are there ongoing challenges you are facing?
   3. What challenges do you anticipate as you continue to establish your platform?
3. Additional support that would assist you in continuing to successfully establish your platform.
   1. Are there specific webinar topics that would help build your platform’s collective capacities?
   2. Are there resources or supports that would help you stay informed about and apply best practices, (i.e., literature, tools, consult calls, etc.)?
4. Capacity building platform participation
   1. Is there anyone else you would like to add to the platform?
   2. Would you like to invite additional members to the next webinar? (Up to 10 people from each platform can attend)
5. Update on deliverables for the WHO Alliance

# Appendix 7: Sample of Platform Monitoring Form

| Monitoring of ERA platforms (team building, stakeholder engagement and demand, rapid products, capacity building, other progress): | | | | | |
| --- | --- | --- | --- | --- | --- |
| building an ERA platform team (describe team recruitment, team structure, and roles/responsibilities of team members) | connecting with policymakers and generating demand (how have you been engaging with policy-makers? what steps have you taken to generate demand for rapid products?) | creating rapid products (list the rapid review products produced and in progress; complete the tabs labeled RES #X for each rapid product listed here) | completing capacity building modules/webinars  (include how many team members are participating in the TAC capacity building activities and to what extent - how many modules/webinars completed?) | other progress (describe other team progress not captured thus far) | Comments: (include any information - challenges, barriers, political context, etc. - that will help clarify progress to date) |

| Monitoring of rapid products (production, stakeholder engagement, dissemination of findings, policy/system influence and impact, and capacity strengthening activities):  nation of findings, policy/system influence and impact, and capacity strengthening activities): | | | | | | | | | | | | |
| --- | --- | --- | --- | --- | --- | --- | --- | --- | --- | --- | --- | --- |
| Title: | | | | | | | | | | | | |
| Objective: | | | | | | | | | | | | |
| Type of Rapid Evidence Product: | | | | | | | | | | | | |
| Commissioning Stakeholder: | | | | | | | | | | | | |
| Start and End Date: | | | | | | | | | | | | |
| month of update | description of methods *(process and tailored methods used)* | policy and/or system issue addressed  *(decision-maker need)* | stakeholders  *(including agency and role, e.g. commissioning agency, collaborating researcher, policymaker, etc)* | stages of stakeholder engagement  *(at what stages of the process was each stakeholder engaged)* | facilitators and barriers to stakeholder engagement  *(include barriers, facilitators, challenges and successes to engaging stakeholders)* | timeliness  *(time taken to respond to decision-maker generated question)* | dissemination methods  *(to ensure uptake of rapid products to policy and decision-making bodies)* | stakeholder perception/satisfaction *(include a number score from stakeholders and any qualitative comments - what they liked/didn't like)* | impact of rapid product  *(on health policymaking and health system strengthening; how was the product used)* | TAC support received *(specify training, consultations, support received and by whom - how many team members)* | process of generating demand for rapid products *(including strategies that worked/didn't work)* | Comments: *(include any unanticipated barriers/challenges and how they were dealt with)* |
| *Month- Year* | *A comprehensive search of the medical and legal databases, websites and reference lists of relevant studies were performed within the review scope.  Study selection, data abstraction and quality appraisal were performed by two independent reviewers to minimise subjectivity and random errors.  The streamlined steps followed in this review included limiting: the study design to randomised clinical trials (RCTs), search dates to a period of 10 years and language of publication to English.* | *Due to an exponential increase in litigation claims related to patient safety in obstetrical care in South Africa, WHO South Africa-Country Office commissioned a review of patient safety initiatives.* | *WHO - South Africa county office (commissioning agency)* | *To ensure the relevance of our review, commissioners from WHO South Africa-Country office were engaged in defining the review scope, developing review questions, approving the protocol and literature search strategies, and identifying key messages.* | *In order to provide decision-makers with timely results, a rapid review approach was collectively agreed upon. This agreement included negotiation of timeline, inclusion criteria and search strategy. The time taken to come to a consensus on these items prior to the start of the review was longer than expected, but it ensured that the review scope and timeframe were suitable to the commissioning agency.* | *6 weeks to provide results to decision-makers* | *Report submitted to WHO decision-makers   Rapid review published in open-access journal and shared widely by the commissioning agency  Conference presentation* | *Score on 1-5 likert scale = 4.2  Decision-maker was satisfied with results as they directly addressed the question and could be used to inform policy change. They felt the submitted report was easy to understand and formatted. They suggested we provide more details on the types of interventions in an appendix.* | *The review found some quality improvement strategies to be more effective for improving obstetrical patient safety than others   Report submitted to WHO decision-makers within 6 weeks used to inform policy development regarding litigation for obstetrics in South Africa* | *Webinars on data selection, abstraction and synthesis (attended by 5 members of the team); Consultations with team regarding appropriate streamlined methods to use* | *We worked on two previous projects with the commissioning agency, and continue to build rapport with them through ongoing collaboration/ engagement, as well as dissemination products targeted to the stakeholders need.* |  |

# Appendix 8: ERA Evaluation Survey

Thank you for participating in the WHO ERA capacity building platform evaluation!

The evaluation survey below will help us measure the process and effectiveness of the World Health Organization’s (WHO) Embedding RApid Reviews in Health Systems Decision-Making (ERA) initiative.

Through this survey, we are asking for your feedback to:

1. Evaluate the effectiveness of the ERA capacity building initiative, i.e., if the initiative supported changes in:

- your knowledge, skills, and confidence to produce rapid evidence synthesis products to meet the needs of knowledge users/decision-makers

1. Evaluate the process of the initiative:

- Whether the initiative was delivered with high quality
- Whether you were satisfied with the initiative offerings
- To gain general feedback on strengths of the program and areas the initiative could be improved

The survey should take no more than 20 to 30 minutes to complete. If you have any questions or technical difficulties, please direct inquiries to Melissa Courvoisier ([Melissa.Courvoisier@unityhealth.to](mailto:Melissa.Courvoisier@unityhealth.to)).

### Participant information questions

1. Please select your ERA platform location:
   1. India
   2. Georgia
   3. Malaysia
   4. Zimbabwe
2. Indicate your role in the ERA platform:

## Effectiveness of the ERA initiative

### Health policy and evidence synthesis knowledge and experience questions

Please rate your level of agreement with each statement on a scale from 1 = **strongly disagree** to 5 = **strongly agree**.

1. I currently have a high level of knowledge about evidence synthesis
2. I have experience synthesizing evidence
3. I am confident in my ability to synthesize evidence
4. I currently have a high level of knowledge on how to synthesize health policy and systems evidence
5. I have experience synthesizing health policy and systems evidence
6. I am confident in my ability to synthesize health policy and systems evidence
7. I currently have a high level of knowledge on how to conduct systematic reviews
8. I have experience conducting systematic reviews
9. I am confident in my ability to conduct a systematic review
10. Has your **knowledge** related to health policy and evidence synthesis changed since the start of the WHO ERA initiative? Please include any examples/reasons for this change.

Yes No Unsure

Please explain:

1. Has your **confidence** related to health policy and evidence synthesis changed since the start of the WHO ERA initiative? Please include any examples/reasons for this change that you can think of.

Yes No Unsure

Please explain:

### Rapid evidence synthesis knowledge and experience questions

Please rate your level of agreement with each statement on a scale from 1 = **strongly disagree** to 5 = **strongly agree**.

1. I currently have a high level of knowledge on how to conduct rapid evidence synthesis and/or rapid reviews.
2. I have experience conducting rapid evidence synthesis
3. I am confident in my ability to conduct rapid evidence synthesis
4. Has your **knowledge** related to rapid evidence synthesis changed since the start of WHO ERA initiative? Please include any examples/reasons for this change that you can think of.

Yes No Unsure

Please explain:

1. Has your **confidence** related to rapid evidence synthesis changed since the start of WHO ERA initiative? Please include any examples/reasons for this change that you can think of.

Yes No Unsure

Please explain:

### Engaging end-users in the process of synthesizing evidence questions

Please rate your level of agreement with each statement on a scale from 1 = **strongly disagree** to 5 = **strongly agree**.

1. I currently have a high level of knowledge on how to stimulate demand for rapid evidence synthesis products
2. I have experience stimulating demand for rapid evidence synthesis products
3. I am confident in my ability to stimulate demand for rapid evidence synthesis products
4. I currently have a high level of knowledge on how to negotiate a review question for a rapid evidence synthesis
5. I have experience negotiating a review question for a rapid evidence synthesis
6. I am confident in my ability to negotiate a review question for a rapid evidence synthesis
7. I currently have a high level of knowledge on how to engage with the end-user throughout the review process
8. I have experience engaging with the end-user throughout the review process
9. I am confident in my ability to engage with the end-user throughout the review process
10. Has your **knowledge** related to engaging end-users changed since the start of WHO ERA initiative? Please include any examples/reasons for this change that you can think of.

Yes No Unsure

Please explain:

1. Has your **confidence** related to engaging end-users changed since the start of WHO ERA initiative? Please include any examples/reasons for this change that you can think of.

Yes No Unsure

Please explain:

1. Have you/your platform experienced any barriers (challenges) when conducting rapid evidence synthesis, developing rapid products, or engaging knowledge users in the process?

Yes No Unsure

Please explain:

1. Have you/your platform experienced any facilitators (supports) when conducting rapid evidence synthesis, developing rapid products, or engaging knowledge users in the process?

Yes No Unsure

Please explain:

## Process of the initiative:

In this section, you are presented with a series of statements related to your satisfaction of each component of the WHO ERA capacity building initiative.

Please rate your level of agreement with each statement on a scale from 1 = **strongly disagree** to 5 = **strongly agree**.

### Inception workshop

Did you attend the inception workshop in Kuala Lumpur, Malaysia on July 17-19, 2018?

- Yes
- No

*Note: If respondents select yes, the next questions below about the inception workshop will be presented. If no is selected, respondents will be taken directly to the online capacity building platform questions.*

- - - 1. Overall, I enjoyed learning at the inception workshop.
      2. The content presented at the inception workshop was relevant to my role/projects.
      3. The inception workshop content was well organized.
      4. There were adequate opportunities for interaction during the inception workshop.
      5. Inception workshop presenters were engaging.
      6. Inception workshop presenters were knowledgeable.
      7. Inception workshop presenters inspired me to learn more.
      8. Overall, I thought the inception workshop was effective in supporting my knowledge and skill development.

Please use the space below to share any additional feedback about the inception workshop:

|  |
| --- |

### Online Capacity Building Platform

1. Overall, I thought the online learning platform was effective in supporting my knowledge and skill development.
2. The content shared on the online learning platform was relevant to my role/projects.
3. The online learning platform was well organized.
4. The resources on the online learning platform were easy to access.
5. There were adequate opportunities for interaction on the online learning platform.

Please use the space below to share any additional feedback about the online Capacity Building platform:

|  |
| --- |

### Online webinars

1. Overall, I enjoyed learning from the online webinars.
2. Overall, the webinar topics were relevant to my learning needs.
3. Overall, the content presented in the webinars was relevant to my role/projects.
4. Overall, the webinars were well organized.
5. Overall, there were adequate opportunities for interaction during the webinars.
6. Overall, webinar presenters were engaging.
7. Overall, webinar presenters were knowledgeable.
8. Overall, webinar presenters inspired me to learn more.
9. Overall, I thought the online webinars were effective in supporting my knowledge and skill development.

Please use the space below to share any additional feedback about the online webinars:

|  |
| --- |

### In-country workshop

1. Overall, I enjoyed learning through the in-country workshop.
2. The content presented at the in-country workshop was relevant to my role/projects.
3. The in-country workshop content was well organized.
4. There were adequate opportunities for interaction during the in-country workshop.
5. The in-country workshop presenters were engaging.
6. The in-country workshop presenters were knowledgeable.
7. The in-country workshop presenters inspired me to learn more.
8. Overall, I thought the in-country workshop was effective in supporting my knowledge and skill development.

Please use the space below to share any additional feedback about the in-country workshop:

|  |
| --- |

### Consultations and additional support

1. Overall, I found the consultations and additional support helpful in supporting my knowledge and skills development.
2. I felt that I could easily request and receive additional support from the TAC team.
3. Overall, the additional resources, feedback, etc., shared by the TAC team was helpful and relevant to my learning needs.

Please use the space below to share any additional feedback about the consultations and additional support provided by the TAC team:

|  |
| --- |

### Additional feedback on the ERA initiative

1. How has your platform’s participation in the WHO ERA initiative impacted health policy and system-level decision making?

|  |
| --- |

1. What did you find to be the most useful part of the WHO ERA initiative?

|  |
| --- |

1. What did you find to be the least useful part of the WHO ERA initiative?

|  |
| --- |

1. Do you have any suggestions for how this initiative could be improved? This will help improve future training initiatives that the Alliance for Health Policy and Systems Research supports.

|  |
| --- |

1. Are there any additional general comments/feedback you would like to provide?

|  |
| --- |

# Appendix 9: LMIC Platform Outputs

| **Country** | **Project** | **Requestor / Knowledge Users** | **Product** | **Usage and Impact** |
| --- | --- | --- | --- | --- |
| Georgia | Pharmaceutical Pricing Policies | Requestor:   - The Parliament of Georgia - Health and Social Issues Committee (HSIC)   Knowledge Users Engaged:  Review scope, questions:   - The Parliament of Georgia, Ministry of Internally Displaced Persons from the Occupied Territories, Labour, Health, and Social Affairs of Georgia (MoILHSA) – State Health Programs - National Center for Disease Control and Public Health (NCDC) - Non-Communicable Disease Department   Literature search strategies   - HSIC | Rapid Review  (22 studies)  Turn-around:  6 weeks  Team members - 2 + 1 policymaker | Rapid response product (‘*Pharmaceutical pricing policies to improve the population’s access to pharmaceuticals in Georgia*’) was shared as a background document for a policy meeting in December 2019 for development of recommendations.  The document was posted on Policy Information Platform (PIP) and shared via social media and email to key parties.  <http://curatiofoundation.org/wp-content/uploads/2022/06/Pharmaceutical-pricing-policies_FINAL.pdf>  <http://curatiofoundation.org/wp-content/uploads/2022/06/Pharmaceutical-pricing-policies_FINAL.pdf>  Several meetings organized by the Parliament of Georgia. Pharmaceutical pricing policies under discussion. |
|  | Pay for Performance for Private Healthcare Providers | Requestor:   - The Parliament of Georgia - Health and Social Issues Committee (HSIC)   Knowledge Users Engaged:  Review scope, questions   - HSIC, meetings with wide group of knowledge users | Evidence Synthesis  (11 SRs, 16 studies)  Turn-around:  15 weeks (delays due to COVID-19)  Team members - 2 + 1 policymaker | Evidence synthesis (‘Effects of Pay for Performance on utilization and quality of care among Primary Health Care providers in Middle and High-Income countries‘) shared with policy-makers involved in primary health care reform. The document posted on Policy Information Platform (PIP) and shared via social media.  <http://curatiofoundation.org/effects-pay-performance-utilization-quality-care-among-primary-health-care-providers-middle-high-income-countries/>  Specific impact has not yet been determined. |
| India | Mid-level Healthcare providers for primary healthcare at Health & Wellness Centres | Requestor:   - National Health Systems Resource Centre (NHSRC)   Knowledge Users Engaged:  Results and implementation   - Report shared at a national consultation with 10 state health system resource centres (SHRCs) and 4 innovation and learning centres (ILCs), Indian Council of Medical Research (ICMR), and the NHRSC. Feedback provided on implementation. | Rapid Policy Brief (12 domains reviewed)  Turn-around:  5 weeks | The policy brief has been posted on the George Institute website and shared via social media:  <https://www.georgeinstitute.org.in/rapid-policy-brief-mid-level-health-providers-mlhps-for-primary-healthcare>  The finalized work was peer reviewed and published: <https://www.ncbi.nlm.nih.gov/pmc/articles/PMC8237370.2/> (May 2021) |
|  | Breast Cancer Screening Using Angle of Tri-Radius (ATD) Measurement | Requestor:   - State Health Systems Resource Centre (SHSRC), at the Atal Bihari Vajpayee Institute of Good Governance and Policy Analysis (AIGGPA), Madhya Pradesh   Review scope, questions   - AIGGPA | Rapid Policy Brief – Narrative Review (0 studies eligible)  Turn-around:  6 weeks | The policy brief has been posted on the George Institute website:  <https://www.georgeinstitute.org.in/rapid-evidence-synthesis-res-on-palmer-angle-tri-radius-for-breast-cancer-screening-in-women>  Abstract accepted for conference presentation. |
|  | Improving dialysis outcomes in chronic kidney disease patients. | Requestor:   - Directorate of Medical Education, Government of Andhra Pradesh   Knowledge Users Engaged:  Protocol, review questions, search strategy   - Government of Andhra Pradesh | Rapid Policy Brief  Turn-around:  16 weeks (delays due to COVID-19)  Team members - 6 + 1 policymaker | A rapid policy brief titled (‘*Health Systems Interventions to Improve Dialysis Outcomes in Patients with Chronic Kidney Disease on Hemodialysis’*) was accompanied by a technical supplement.  Impact yet to be determined, as the draft brief was awaiting feedback. |
|  | Best practice training module for attendants providing maternal postnatal care including nutrition and breastfeeding | Requestor:   - The District Medical Officer (DMO), Malappuram, Kerala   Knowledge Users Engaged:  Review scope, questions   - The District Medical Officer (DMO), Malappuram, Kerala | Rapid Policy Briefs  Turn-around:  7+ weeks (delays due to COVID-19)  Team members - 5 + 1 policymaker | 3 rapid policy briefs and 3 technical supplements addressing 3 components. Titles of policy briefs (draft as of May 2020):  1. Summary of Recommendations from Relevant Guidelines on Best Practices for Postnatal Care  2. Harmful Postpartum Beliefs and Practices of Mothers in India  3. Training of Post-Natal Care Attendants for Post-Natal Care, Nutrition and Breastfeeding.  <https://www.georgeinstitute.org.in/training-of-post-natal-care-attendants-for-post-natal-care-nutrition-and-breastfeeding-rapid-policy> (August 2020) |
|  | Improving diagnosis, quality of care, and prevention methods for asthma and COPD | Requestor:   - State Health Resource Centre (SHRC), Chhattisgarh   Knowledge Users Engaged:   - SHRC | Rapid Policy Briefs  Turn-around:  7+ weeks (delays due to COVID-19)  Team members - 4 + 1 policymaker | SHRC requested evidence-informed policy recommendations for a LMIC primary care context:  1. Improving diagnosis of COPD  2. Improve Quality of Care (QoC) in children and adolescents with asthma and adults with COPD  3. Primary prevention of asthma and COPD.  Several policy briefs and technical supplements were provided, with some still to be completed. Dissemination has not yet occurred. |
| Malaysia | Midwife Competency/ Qualification for Antenatal Care | Requestor:   - Family Health Development Division, Ministry of Health Malaysia   Knowledge Users Involved:  Review question, Search Strategy, Preliminary findings:   - Family Health, Ministry of Health - Nursing Division, Ministry of Health - Training Division, Ministry of Health - Maternal Health Service Providers | Rapid Evidence Synthesis Report (63 documents included)  Turn-around:  5 months  Team members – 10 + 4 academic | All MAera projects, reports and output can be found in the MAera online platform at: <https://maera.nih.gov.my/index.php>  Final report (‘Midwifery Qualification in Primary Care’):  <https://maera.nih.gov.my/index.php/component/advportfoliopro/project/53-rapid-evidence-synthesis-on-midwifery-qualification-in-primary-care?catid=84:completed-projects&Itemid=437>  The review found that most countries with better maternal mortality rates had midwives with degree-level education. Directors (stakeholders) used the report to argue for continued support of the previously approved plan of piloting degree-level education for nurses despite the change in Health Minister. |
|  | Outcome Indicators for Pressure Injury | Requestor:  Nursing Division, Ministry of Health  Knowledge Users Involved:  Review scope, questions, search strategy, data points, Preliminary Findings   - Nursing Division, Ministry of Health - Patient Safety Unit, Ministry of Health - Family Health Development Division - Training Management Division, Wound Care Unit, Hospital Kuala Lumpur   Data extraction   - Nursing Division, Ministry of Health | Policy Brief, (Rapid Evidence Synthesis Report)  Turn-around:  10 months  Team members – 17 + 1 academic | Review will be used to re-design the definition of hospital-acquired pressure injury for Malaysia. Nursing Division plans to review and update the national guideline. A national pilot project using the new indicators is planned after the review. IHSR Director promoted MAera services to knowledge users and the Medical Development Division at the final knowledge user presentation.  <https://maera.nih.gov.my/index.php/component/advportfoliopro/project/51-outcome-indicators-for-monitoring-of-pressure-injury-prevention-report?catid=84:completed-projects&Itemid=437>  (Technical report published March 2022) |
|  | Models for Integrative Medicine to include Traditional and  Complementary Medicine (T&CM) | Requestor:   - Tradition(T&CM) al & Complementary Medicine Division, Ministry of Health   Knowledge Users Involved:  Review scope, search strategy   - T&CM, pharmacists, Medical Officers | Rapid Review (found 12 frameworks)  Turn-around:  6 months  Team members – 6 + 2 Medical Officers, 2 pharmacists | Findings will inform the development of a national integrative medicine framework.  The review did not find any published solid framework for integrative medicine. Further discussion with policy-makers and appropriate knowledge users is needed to consolidate the application of review findings for decision-making.  https://maera.nih.gov.my/index.php/component/advportfoliopro/project/56-report-for-rapid-evidence-synthesis-on-approaches-to-integrative-medicine?catid=84:completed-projects&Itemid=437 |
|  | Population Wellbeing Outcomes Framework | Requestor:   - Public Health Development Division (Policy and Public Health Services)   Knowledge Users Involved:  Review scope, questions, Preliminary Findings:   - Public Health Development Division (Policy and Public Health Services) | Rapid Review Brief  Turn-around:  10 days  Team members – 7 + 2 policymakers | The review found 12 frameworks for population well-being outcomes that were used to inform the 5-year Twelfth Malaysia Plan. Review produced indicators that could be applied to current national health indicators. Two members from MAera were invited to the technical working group for Improving Population Health Outcomes to prepare for the Twelfth Malaysia Plan. Findings were adapted into the framework proposed for the Plan.  <https://maera.nih.gov.my/index.php/index.php?option=com_advportfoliopro&view=project&id=3:portfolio-3&catid=84:completed-projects> |
|  | Telemedicine and Patient Satisfaction | Requestor:   - Infectious Diseases team at Hospital Sg. Buloh (referral hospital for infectious diseases in Malaysia)   Knowledge Users Involved:  Review scope, questions, search strategy:   - Infectious Diseases team at Hospital Sg. Buloh (referral hospital for infectious diseases in Malaysia) | Rapid Review  Turn-around:  4 months  Team members – 7 + 1 policymaker | Patient satisfaction tools were extracted from the review inventory and presented to the knowledge users.  <https://maera.nih.gov.my/index.php/component/advportfoliopro/project/54-patient-satisfaction-tools-in-telemedicine-a-maera-rapid-evidence-inventory?catid=84:completed-projects&Itemid=437> |
|  | Integrated Care Measurement | Requestor:   - National Head of Service for Internal Medicine   Knowledge Users Involved:  Review scope, questions, Preliminary Findings:   - National Head of Service for Internal Medicine | Rapid Review (Brief report – ongoing)  Turn-around:  4 weeks (ongoing)  Team members – 8 + 2 policymakers | The list of instruments will be used to discuss with knowledge users the best approach to measure care integration. A study proposal, commissioned by the same stakeholder, is currently being developed using the findings of this rapid review to measure interdisciplinary collaboration among doctors in an inpatient setting.  <https://maera.nih.gov.my/index.php/component/advportfoliopro/project/38-integrated-care-measurement-intecare?catid=84:completed-projects&Itemid=437> |
| Zimbabwe | Enrolment of the informal sector into the national health insurance (NHI) program | Requestor:   - Chief Director for Policy and Planning, Monitoring and Evaluation, Ministry of Health and Child Care (MoHCC)   Knowledge Users Involved:  Review scope, questions:   - MoHCC - Ministry of Finance and Economics - Cabinet - Development Partners - Ministry of Public Service - Labour and Social Welfare   Review question   - MoHCC | Rapid Review – Policy Brief (9 studies included)  Turn-around:  8 weeks  Team members – 14 + 1 policymaker  Score =4/5 | The Minister of Health and Child Care received the report and presented a paper to Cabinet. A draft bill for the National Health Insurance is now being developed.  This review appears to have generated new requests of the team.  Enrollment and contributions to national health insurance should consider gender, age, demographic status (urban/rural, marital status), education and health status.  <https://www.zeipnet.co.zw/node/12> |
|  | National Health Accounts | Requestor:   - Zimbabwe Statistical Agency   Knowledge Users Involved:  Review scope, questions:   - MoHCC | Rapid Review – Policy Brief  Turn-around:  4 weeks  Team members – 8 | Results shared with stakeholders at a Health Financing Symposium. |
|  | Factors affecting the uptake of HIV Self testing among adults in Zimbabwe | Requestor:   - Ministry of Health and Child Care, HIV and AIDS unit   Knowledge Users Involved:  Review scope, questions:   - Ministry of Health and Child Care, HIV and AIDS unit | Rapid Review  Turn-around:  8 weeks  Team members – 14 | *‘Rapid review on the factors affecting the uptake of HIV Self Testing among adults in Zimbabwe’.*  It is anticipated that the rapid review will influence the re-orientation of the current HIV self-testing program so as to improve uptake of HIV self-test kits amongst the adult population |

# Appendix 10: LMIC Platform Outputs Related to COVID-19

| **Country** | **Project** | **Requestor / Knowledge Users** | **Product** | **Usage and Impact** |
| --- | --- | --- | --- | --- |
| Georgia | The COVID-19 epidemic in Georgia Projections and Policy Options | Requestor:   - The Parliament of Georgia, Ministry of Internally Displaced Persons from the Occupied Territories, Labour, Health, and Social Affairs of Georgia (MoILHSA) | Rapid Response Product (25 papers included)  Turn-around:  10 days  Team members – 5 | Report covered widely in the media and social media: <http://curatiofoundation.org/covid-19-epidemic-georgia-projections-policy-options/>. This included modelling the effects of social distancing scenarios: <http://curatiofoundation.org/4-scenarios-covid-19-epidemics-line-social-distancing/>.  Report provided recommendations on planning and coordination, public health actions, health system measures, infection prevention and control measures in medical facilities, as well as scenarios of epidemic progression. Report was delivered to the Prime Minister's office and was a basis for introduction of the lockdown in March.  Subsequently, the team’s Rt (infection reproduction number) was used for monitoring by policy-makers, and recommendations on lifting of measures were also used by the government. ERA platform members were invited to high-level policy decision making meetings with the Prime Minister and were part of the COVID-19 working group with MoILHSA.  Additional support for financing models for COVID-19 hospital case management, and telemedicine healthcare provider payment mechanisms. |
| India | Frontline health workers (FLHWs) in COVID-19 prevention and control | Requestor:   - National Health Systems Resource Centre (NHRSC) | Rapid Evidence Synthesis (policy brief)  Inventory of guidelines and resources for training materials for FLHWs  Turn-around:  3 days  Team members – 5 + 1 policymaker | The brief (<https://www.georgeinstitute.org/frontline-health-workers-in-covid-19-prevention-and-control-rapid-evidence-synthesis>) was used in developing a Ministry of Health & Family Welfare (MoHFW) brochure for India's FLHWs. The brief’s recommendations also informed policy of the Odisha government: community health workers continued to deliver essential services, while a separate cadre conducted field surveillance. Media hailed Odisha a success.  The brief informed the ‘COVID-19 Preparedness Checklist for Rural Primary Health Care & Community Settings’, a collaboration of clinicians and public health researchers from leading institutions in India.  DFID Nepal translated the brief policy recommendations into Nepali. Evidence Aid summarized the brief for their website portal that makes COVID-19 evidence accessible to decision makers. The Institute of Public Health, Bengaluru, listed the brief in its COVID-19 resource inventory. The brief was featured in the National Library of Medicine, NIH portal called Disaster Information Management Research Centre. Two WHO documents referenced the brief.  Organisations and advocacy groups used the brief to support their work or have included it in their resources repository. This includes but is not limited to the COVID-19 PHC Action Group, International Health Policies, EQUINET, Community Health Impact Coalition, and UK Working Group of NCDs. |
| Malaysia | Malaysia's Health Systems Response to COVID-19 | Requestor:  Deputy Director General (Research & Technical Support)  Knowledge Users Involved:  Deputy Director General (Research & Technical Support) | 1. Malaysian health systems response online dashboard  2. Manuscript (draft)  3. Report (planning stage)  Turn-around:  2 months (ongoing)  MAera members - 8 | 1. Online dashboard available for public viewing, refined live.  2. Stakeholder presentation to reviewer commissioner on 18 May 2002.  3. Commissioner will pass on outputs to the Director General of Health and Health Minister.  1) Final report:  <https://maera.nih.gov.my/index.php/component/advportfoliopro/project/39-maera-final-report-june-2020?catid=84:completed-projects&Itemid=43>  2) Link to online MAera dashboard:  <https://maera.nih.gov.my/index.php>  3) Link to journal publication: <https://doi.org/10.3390/ijerph182111109> . |
| Zimbabwe | Mandatory COVID-19 related Institutional Quarantine for Returning Residents in Zimbabwe: A Rapid Response Brief | Requestor:   - National Inter-ministerial Task Force on Covid 19 (MoHCC)   Knowledge Users Involved:   - College of Public Health Physicians of Zimbabwe   Review questions   - MoHCC | Rapid Brief  Turn-around:  2 weeks  Team members – 16 | Rapid evidence brief on the ideal duration of institutional quarantine, - leading to the extension for returning citizens from 14 to 21 days. Brief also provided evidence leading to the requirement that psychosocial services be provided for people under quarantine.  A presentation was made to the COVID-19 National Taskforce and products were shared electronically among decision makers and other stakeholders. The Quarantine Guidelines developed using the rapid product, were also disseminated on the WhatApp platform to many different end users, including Non-Governmental Organisations, Professional Associations, Other government ministries and the general citizenry. |
|  | Use of Face Masks by general public in the COVID-19 Response | Requestor:   - National Inter-ministerial Task Force on COVID-19 (MoHCC)   Knowledge Users Engaged:   - Primary Care Physicians, College of Public Health Physicians of Zimbabwe   Review questions   - MoHCC | Rapid Review  Turn-around:  2 weeks  Team members – 16 | The product was used to inform policy: Cabinet proclaimed a mandatory face covering policy for all citizens when in public. The ERA platform led the drafting of the Guidance for Use of Face Masks in Public. One of the attorneys within the Ministry of Health and Child Care announced at a meeting of Ministry directors that he has used and quoted the guidance document developed from this rapid product in a court of law.  The product was shared with policy makers during a senior management meeting. The guidelines have been disseminated by e-mail and the WhatsApp platform to professional association, NGOs, and civil society organisations. |

# Appendix 11: ERA Evaluation Survey Results

WHO TAC ERA Evaluation Survey Summary

Eleven (11) participants from the four platforms completed the evaluation survey. Survey results are shown below.

### Participant information questions

**Q1. Please select your ERA platform location**

| **Platform** | **Number of survey respondents** |
| --- | --- |
| Georgia | *counts omitted for confidentiality* |
| India |  |
| Malaysia |  |
| Zimbabwe |  |
| **Total** | **11** |

**Q2. What is your role in the ERA platform?**

- Technical Support
- Evidence Synthesis Specialist
- Project Manager
- Librarian
- Student and Reviewer
- Researcher
- Evidence Synthesis
- Lead
- Methods Lead , Rapid Evidence Synthesis
- Platform implementation participant

## Effectiveness of the ERA initiative

### Healthy policy and evidence synthesis knowledge and experience questions

**Q3 - Please rate your level of agreement with each statement on a scale from strongly disagree to strongly agree.**


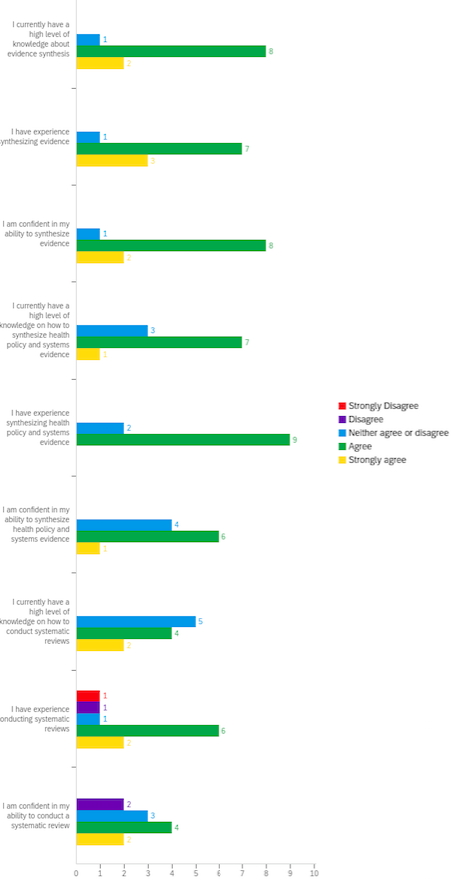


**Q4. Has your knowledge related to health policy and evidence synthesis changed since the start of the WHO ERA initiative?**


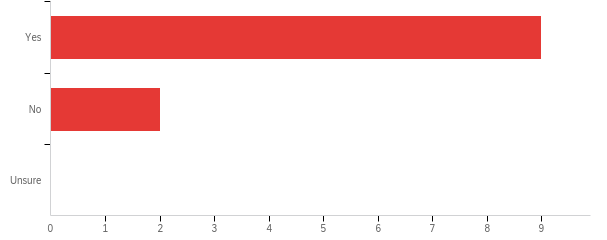


**Please share any examples/reasons for this response.**

- *I now know that it’s possible to institutionalize the evidence into policy rather than leave it to individual initiative*
- *Had some bit of prior knowledge on systematic reviews but not rapid reviews and the WHO ERA initiative imparted some knowledge on rapid reviews*
- *Experience was gained mainly in terms of contexualising local health policy evidence and providing policy options, rather than practice recommendations. Other experience related to an understanding of how to streamline methods for rapid evidence synthesis.*
- *I can now come up with a search strategy , protocol and can do the inclusion and exclusion criteria as a librarian*
- *I have worked through reviewing articles to debrief on policy related issues, thereby allowing for quick decisions to be taken by policy makers, which provided quick answers by building on data that is already available and aligning the available information its the feasibility on the current scenario.*
- *Before the ERA initiative my knowledge related to evidence synthesis was narrowed and it was based on my practical work since I have been working on policy briefs development that entailed local evidence and synthesized international evidence related to local priorities. Introduction to systematic review course was very fruitful for me. It explained all steps and details needed for systematic review development. Before the ERA initiative I did not know all these details related to systematic reviews and other evidence synthesis product development.*
- *There was an increase in knowledge and skills regarding rapid reviews and their critical role in providing research evidence to policymakers in a timely and efficient manner. It was also enlightening to learn that rapid reviews also use systematic review principles and approaches. The webinars were also critical in building skills and knowledge on how to identify relevant context-specific policy issues which needed to been informed by evidence, these skills and knowledge assisted in identifying key policy issues around national health insurance.*
- *In the past, evidence synthesis focused on clinical topics which is more straightforward as the focus in on effectiveness of interventions. Adapting the rapid review methodology in the projects under the MAera platform gave the opportunity to test out feasibility of conducting rapid reviews in the area of health systems in Malaysia. We have found that from the first year of conducting rapid reviews, most of the evidence needs that our policymakers requested for gravitated towards grey literature search.*
- *The capacity building was very primitive in nature and not at all relevant to LMIC context*

**Q5. Has your confidence related to health policy and evidence synthesis changed since the start of the WHO ERA initiative?**


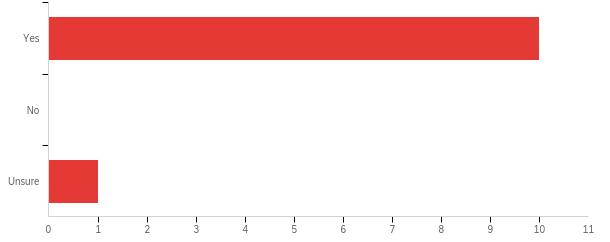


**Please share any examples/reasons for this response.**

- *I know soon there will be a guideline to ensure standard approaches*
- *I would say my confidence improved somewhat but I am still not very confident on certain aspects of systematic reviews*
- *I feel more confident with the use of streamlined evidence synthesis methods, from a health systems and policy perspective.*
- *I am now confident in synthesizing evidence*
- *It has changed because I have seen increased interest of decision makers in using the ERA team in assisting in coming up with decisions on health policies.*
- *Before the ERA project I have only summarized the evidence around different HPSR topics while working on some reports and peer-reviewed publications. But after the ERA project start point I was involved in real evidence synthesis product development. So now I feel confident that I can by myself synthesize HPSR evidence.*
- *The implementation of capacity building initiatives and institutionalisation at three levels, that is (individual, institutional, systemic environment) was key in increasing the confidence levels related to health policy and evidence synthesis. The three-pronged approach was key in strengthening confidence levels.*
- *Although there has been an increase in the level of skills and knowledge, I feel that my confidence level has only slightly changed. This could be due to the different nature of evidence needs from the policymakers in Malaysia, that resulted in grey literature review, which does not fit into the mould of the rapid review of articles retrieved from databases which was taught to us.*

### Rapid evidence synthesis knowledge and experience questions

**Q6 - Please rate your level of agreement with each statement on a scale from strongly disagree to strongly agree.**


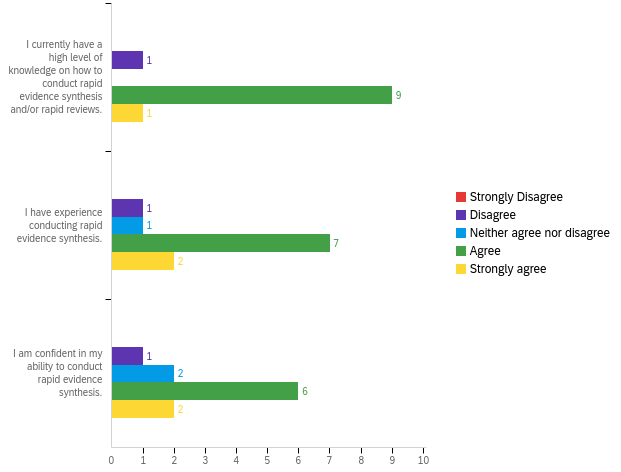


**Q7. Has your knowledge related to rapid evidence synthesis changed since the start of the WHO ERA initiative?**


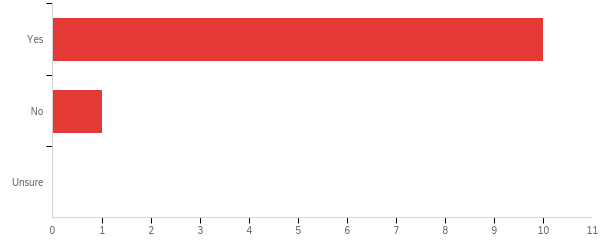


**Please share any examples/reasons for this response.**

- *I need exposure as to how to do it*
- *Refer to previous reasons*
- *The different streamlined methods used on devloping and formulating health systems and policy questions.*
- *I now have knowledge to search through different databases*
- *Yes since I have used it to assist in comimg up with policy decision following our input through policy briefs to policy makers.*
- *I am a researcher at my organization involved in evidence production coming from primary studies. I was rarely involved in knowledge synthesis products development before the start up of ERA project. My knowledge related to rapid evidence synthesis improved since the beginning of ERA initiative as I get used to nee literature about how to conduct rapid reviews. It should also be noted that webinars dedicated to different themes of evidence synthesis steps was very important for me to improve my knowledge towards this direction.*
- *My knowledge related to rapid evidence synthesis process as a dynamic and interactive process has changed. Previously I viewed rapid evidence synthesis as static but after going through the webinars and the ACRES training, I learnt that rapid evidence synthesis is dynamic, intuitive and interactive. I also learnt that specific skills set such as the ability to access, evaluate and synthesize evidence is key in rapid evidence synthesis. However, given the orientation towards health system and policy research, most of the synthesis which we conducted used qualitative approaches. Knowledge and skills on how to use evidence appraisal tools such as AMSTAR 1 and 2 were also novelty and unprecedented.*
- *I am more familiar with streamlining methods for rapid evidence synthesis.*

**Q8. Has your confidence related to rapid evidence synthesis changed since the start of the WHO ERA initiative?**


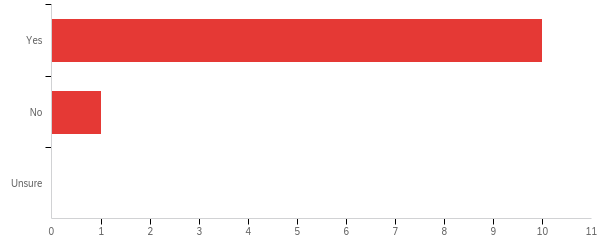


**Please share any examples/reasons for this response.**

- *I await training*
- *Refer to previous reasons*
- *I have been conducting and developing more rapid reviews and rapid policy briefs meeting the relevant policy decision-makers' needs.*
- *Can explore through various databases in search of information.*
- *Yes the grown intrest by policy makers to adopt the ERA team input to their decisions.*
- *It was my first experience to produce rapid evidence synthesis document under the WHO ERA initiative.*
- *As part of a team, I am now able to develop a rapid response question from a policymaker inquiry and determine the scope of topical content, search for research evidence using PICOTS or SPICE and appraise the evidence, then contextualise and summarise the evidence.*
- *Support from the TACs, AHPSR's guide for rapid reviews and our team's search from other sources for documents that could help our work in conducting rapid evidence synthesis raised my confidence as all these provided me with the knowledge required.*

### Engaging end-users in the process of synthesizing evidence questions

**Q9. Please rate your level of agreement with each statement on a scale from strongly disagree to strongly agree.**


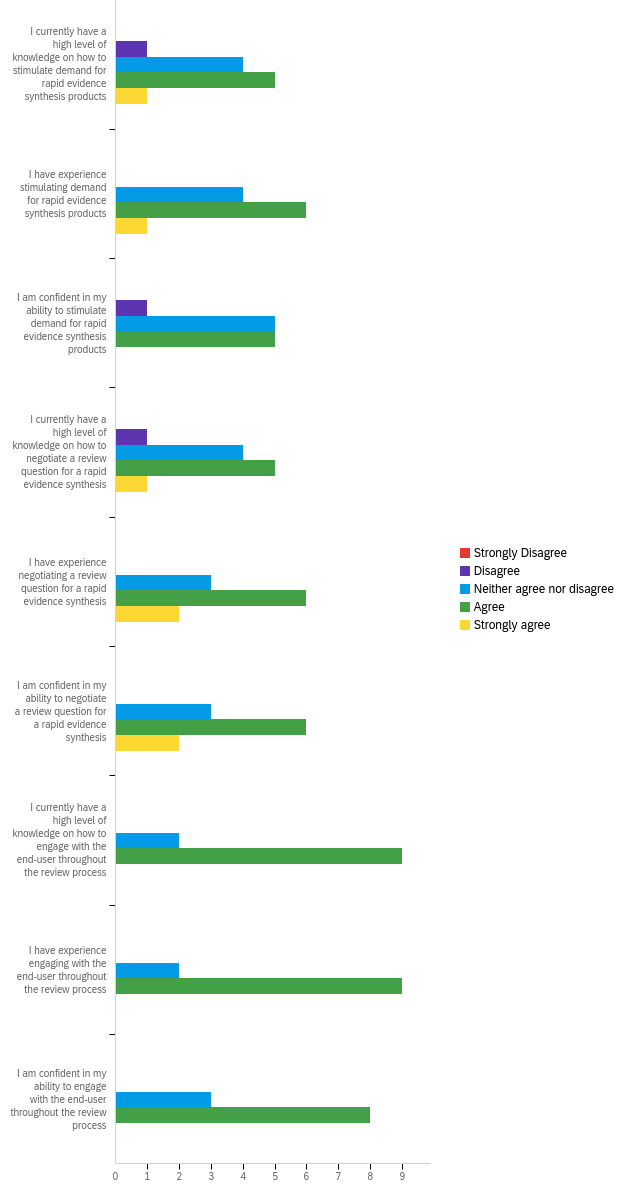


**Q10. Has your knowledge related to engaging end-users changed since the start of the WHO ERA initiative?**


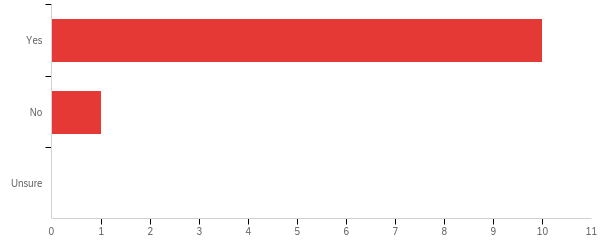


**Please share any examples/reasons for this response.**

- *Not had the opportunity to review nor practice*
- *We have been able to successfully obtain high level buy-in and increased demand for rapid reviews by policymakers within the ministry and other stakeholders*
- *Working on a few rapid evidence synthesis products helped gain an understanding of stakeholder's perspectives and their interest in engaging throught the synthesis process.*
- *Can clearly conduct a reference interview to end users in refining the queries*
- *Yes more and more often we have had to go to the end user to get clarification to their research questions. This engagement has made it possible that we review in line with the need of the end user.*
- *During working on the rapid evidence synthesis product about pharmaceutical pricing policies in Georgia I had to negotiate research question to policy making institution - end-user of this product. Using email communication in conjunction with face to face meetings we achieved to engage this institution throughout the product development.*
- *We have used mostly high-level management meetings to stimulate demand and engage end-users. ERA platform is now automated and this is key in stimulating demand and engaging end-users. One of the key lessons which were learnt was that there has to be an active demand side and continuous stimulation for the demand for evidence. Sensitisation meetings, presentations were some of the tools which were used to stimulate demand for the ERA products. However, as the service progresses, the demand needs to be sustained through regular engagement between the ERA platform and it's users.*
- *Under the platform we have tested out various methods of stakeholder/policymaker involvement in rapid evidence synthesis. The experience in co-creation of knowledge, which is the crux of rapid reviews, has increased knowledge and skills in engaging with the end users*

**Q11. Has your confidence related to engaging end-users changed since the start of the WHO ERA initiative**?


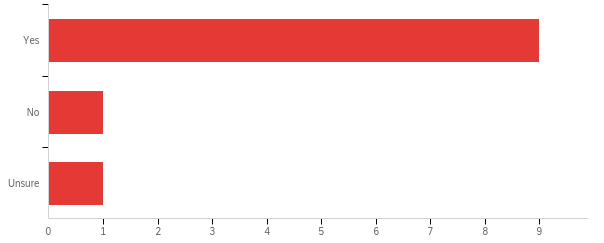


**Please share any examples/reasons for this response.**

- *I’m still to get an opportunity to put into practice*
- *I can confidently engage and articulate the importance of rapid reviews and the process of coming up with these including the roles of various stakeholders*
- *Increasingly, with more rapid evidence syntheses undertaken, the process of negotiating with the end-users has been on the 'up'.*
- *I now have experiences in refining queries*
- *I find it easier to navigate further with clear knowledge of what the end user requires.*
- *The previous experience of engaging end-users in evidence synthesis product development increased my confidence how to do this in practice.*
- *More efforts need to be put on activities that promote and facilitate continuous engagement of end-users and stimulating demand for the rapid evidence products and services. Moreso, as the service progresses the demand needs to be sustained. There is also a need for sustainability activities on the building/stimulating demand for the use of knowledge translation platforms post the WHO ERA initiative.*
- *Under the platform we have tested out various methods of stakeholder/policymaker involvement in rapid evidence synthesis. The experience in co-creation of knowledge, which is the crux of rapid reviews, has increased knowledge and skills in engaging with the end users*

### Barriers and facilitators

**Q12. Have you/your platform experienced any barriers (challenges) when conducting rapid evidence synthesis, developing rapid products, or engaging knowledge users in the process?**


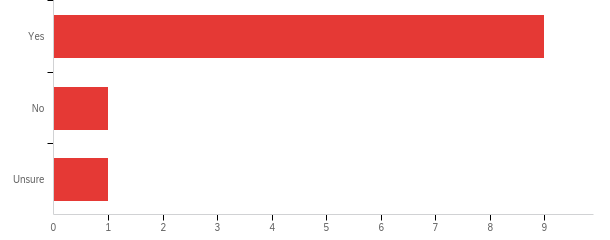


**Please explain.**

- *Perhaps I need an opportunity to put into practice*
- *Some barriers that we have encountered include: - Timely production of rapid evidence products due to government bureaucracy - Availability of ERA team members due to other work commitments as these were not sorely delegated to the platform activities - Balancing quality of evidence products and urgency of producing rapid evidence products - Challenges in framing rapid review questions mainly due to failure by the policymakers to adequately articulate what evidence they really required - Limited engagement of end-users because of lack of availability of policymakers during the rapid review process*
- *Mainly with guiding stakeholders towards appropriate review questions, and to a certain extent getting timely feedback during the various stages of the synthesis process.*
- *The finalization of the research question for rapid response product took longer than planned as policymakers requested for very wide topic at the beginning.*
- *The Ministry and government are supportive*
- *End users were at time not too sure of their clear requirement only to realign and affirm their needs with further probing from the ERA platform members.*
- *In order to provide decision-makers with timely results, a rapid response product approach was agreed upon at a later stage. Due to the broad nature of the review question it was a challenge for the review team to narrow down the original research question. The time taken to come to a consensus on these items prior to the start of the review was longer than expected as well as the process of 1st rapid response product development.*
- *One of the key challenges/barriers, when conducting rapid evidence synthesis or developing rapid products within a Government Ministry of Health is that the policymaking process is inherently a political process as there are many stakeholder interests influencing policymakers. This was evident during the stakeholder engagement process of the National Health Insurance Rapid Review, where policymakers from different Government Ministries such as Finance, Gender, wanted their views or perceptions to be included in the rapid review. However, some of the evidence provided did not meet the necessary criteria to be included in the rapid review product. One of the key challenges of the ERAZ initiatives in the early stages was on building trust between the policymakers and the evidence synthesis products producers. However, as the programme evolved the ERAZ team members gain traction and gradual trust from the policy makers.*
- *Challenges in conducting rapid evidence synthesis: 1. Deciding on source of articles/documents - conducting a database review first or proceeding straight to document review from grey literature 2. Getting the policymakers to identify their intention of commissioning the rapid review 3. Quality appraisal of documents retrieved through grey literature Engaging knowledge users in the process: 1. Preference for face-to-face meetings over phone/skype meetings 2. Time constraints*

**Q13. Have you/your platform experienced any facilitators (supports) that could be utilized when conducting rapid evidence synthesis, developing rapid products, or engaging knowledge users in the process?**


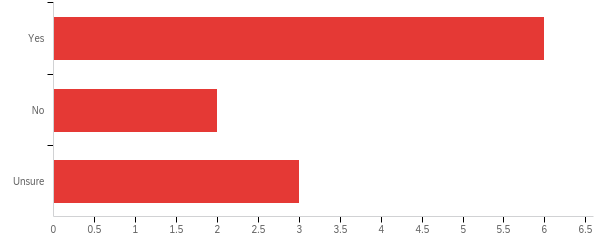


**Please explain.**

- *Still to experience*
- *Some of the facilitators include: - High level buy-in and political support - Ensuring the platform obtains a budget line and is part of the institutional annual workplans - Capitalising on health strategic plans and ensuring the platform has a pervasive role in generating evidence to support implementation - Involvement of key departments, for example library - Contextualised training*
- *More technical know-how through webinars, and through experience!*
- *The topic of the pharma price regulations was very important for the country which was facilitator in developing the rapid response product*
- *Known by the team leaders*
- *Facilitator of engagement of different stakeholders was the high interests related to priority of the topic. The main barrier observed was busy political environment in country related genral political issues linked with election policy formulation. Which resulted in shortened period of policy makers presense on the meetings.*
- *One of the key facilitators that facilitate conducting rapid evidence synthesis is having leadership that supports and promotes the use of evidence and buy-in from the Minister's and Permanent Secretary's office*

## Process of the initiative:

In this section, participants were presented with a series of statements related to their satisfaction of each component of the WHO ERA capacity building initiative.

### Inception Workshop

**Q14. Did you attend the inception workshop in Kuala Lumpur, Malaysia on July 17-19, 2018?**


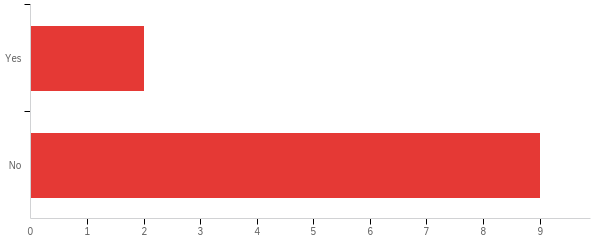


**Q15. Please rate your level of agreement with each statement on a scale from strongly disagree to strongly agree.**


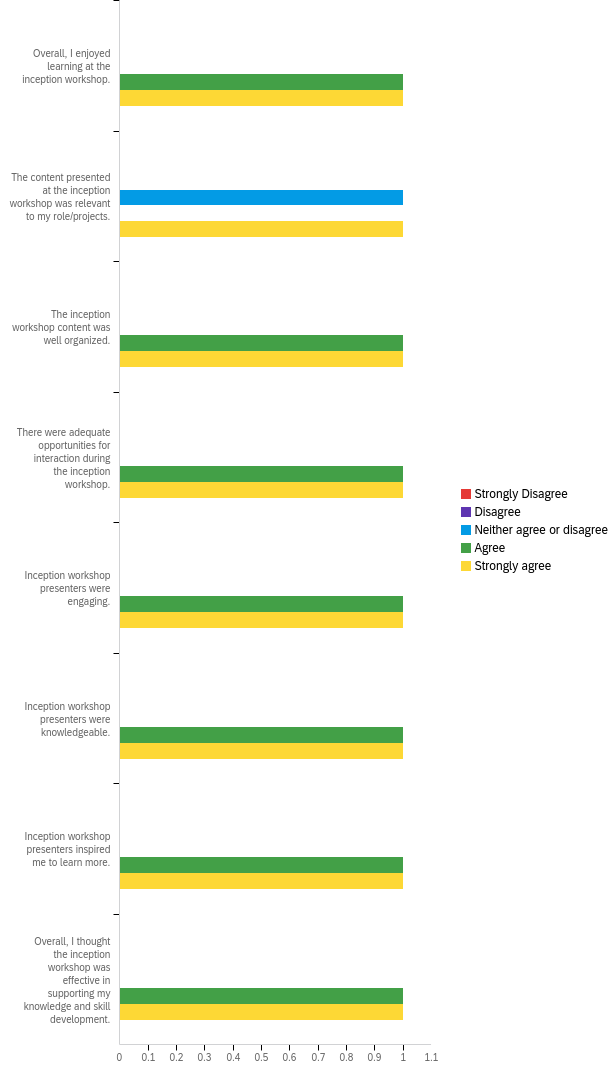


**Q16. Please use the space below to share any additional feedback about the inception workshop:**

- *More content on conducting rapid reviews and the problems that reviewers may face when conducting rapid reviews should be presented*

### Online capacity building platform

**Q17Please rate your level of agreement with each statement on a scale from strongly disagree to strongly agree.**


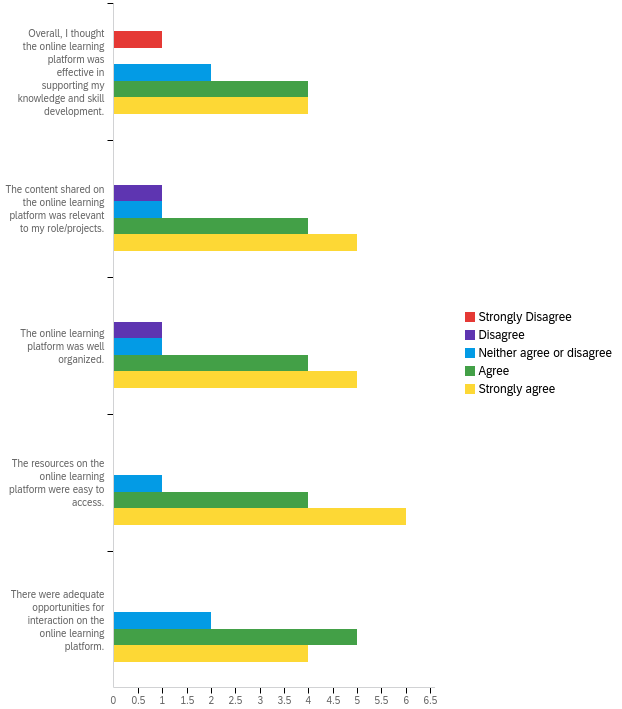


**Q18. Please use the space below to share any additional feedback about the online capacity building platform:**

- *Was not able to participate*
- *I think some of the content was too technical especially for starters and the training also lacked some of the more softer training content required in embedding or institutionalising rapid evidence synthesis platforms*
- *More encouragement for different platforms to interact and engage with.*
- *The online building capacity was very useful*
- *The online platform was useful in corroborating the literature and putting content into practice.*
- *Things that would be more helpful: 1. Open discussion on problems faced by platforms - we may have faced the same problems and could probably troubleshoot for solutions together.*

### Online webinars

**Q19. Please rate your level of agreement with each statement on a scale from strongly disagree to strongly agree.**


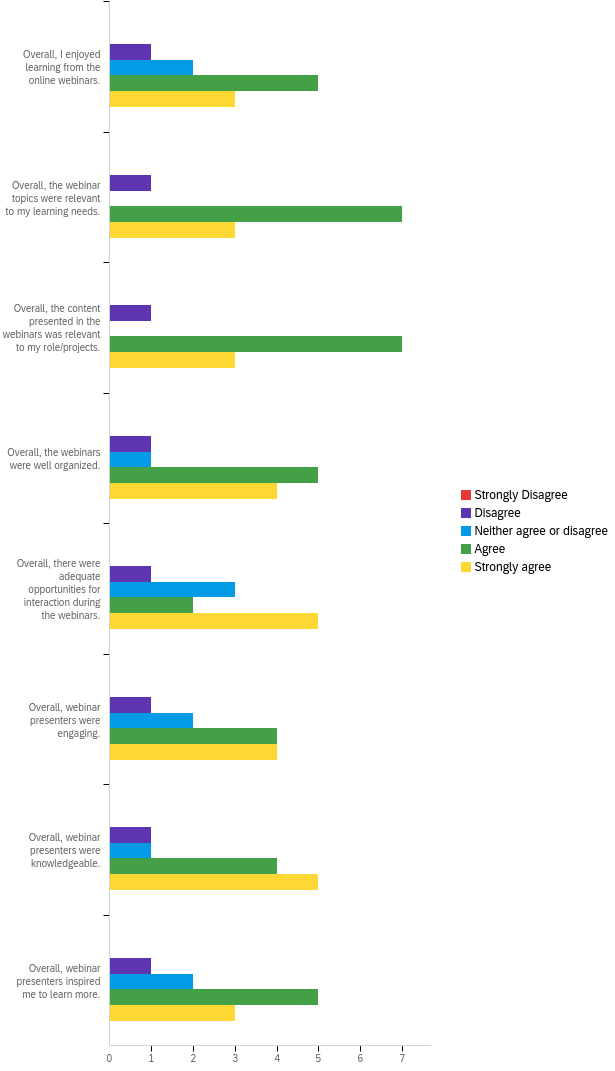


**Q20. Please use the space below to share any additional feedback about the online webinars:**

- *The webinars were very informative and very useful due to the fact that one could replay the webinars again and again until one understands the concept.*
- *Very useful continuous learning and knowledge sharing platform*
- *(Same as previous comment)*

### In-country workshop

**Q21. Did you attend the in-country workshop?**


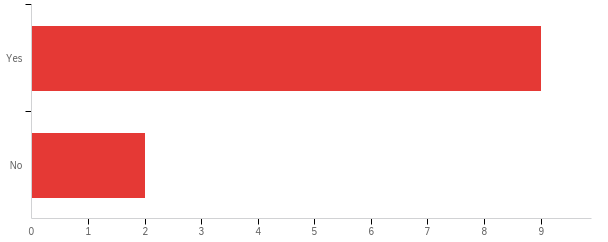


**Q22. Please rate your level of agreement with each statement on a scale from strongly disagree to strongly agree.**


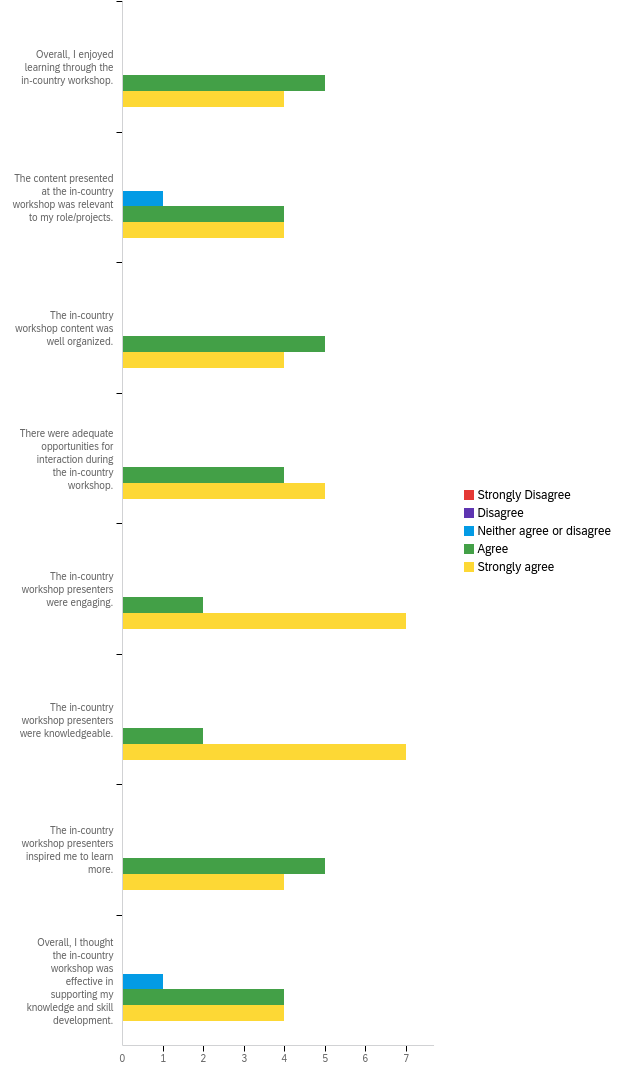


**Q23. Please use the space below to share any additional feedback about the in-country workshop:**

- *The in-country workshop was more contextualised, the facilitators were more practical and used real life examples based on practical experience.*
- *NA*
- *The presenters were knowledgeable and used examples to demonstrate which was good*
- *The in-country workshop demystified concepts and processes in using rapid response mechanism*
- *In retrospect, more content on searching for grey literature search and guide on deciding on the source of information (database versus other sources) would facilitate conduct of rapid evidence synthesis.*

### Consultations and additional support

**Q24. Please rate your level of agreement with each statement on a scale from strongly disagree to strongly agree.**


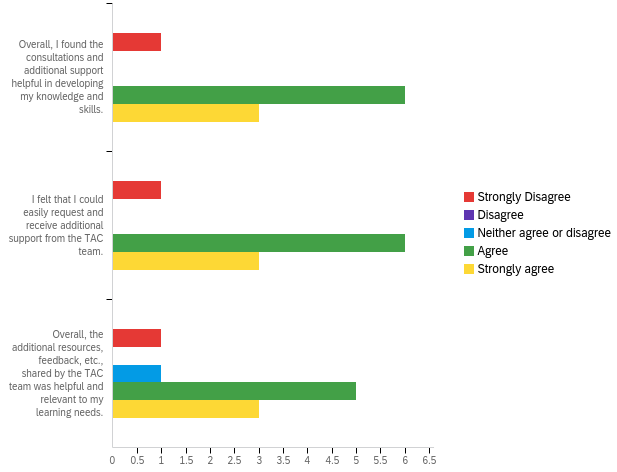


### Additional feedback

**Q25. Please use the space below to share any additional feedback about the consultations and additional support provided by the TAC team:**

- *Due to work commitments, I missed out on the meetings and trainings. The additionals have allowed me to keep abreast of what is happening.*
- *The TAC Team was available to support and were so open and approachable but I do not think our ERAZ Platform utilised the team as much as they should have done. I think the TAC team was heavily underutilised.*
- *NA*
- *More support may be required to equip the teams with more skills*
- *I am very grateful to TAC team! They did additional work for us by reviewing search strategy and final product produced by our team.*
- *TAC team did not seem to have LMIC relevant experience. Might have been better if Ugandan colleagues hosted it.*

### Impact of platform

**Q26. How has your platform’s participation in the WHO ERA initiative impacted health policy and system-level decision making?**

- *Not sure*
- *Yes, it has informed the national health strategy mid term review and also the National Health Insurance Bill to be tabled before Parliament*
- *Work in-progress*
- *The product developed in the frame of the ERA platform informed policymakers how to change the pharma pricing policy and the decision has been made to consider the recommendations while changing the policy.*
- *It impacted so much for instance our first RR on Uptake of compulsory National Health Insurance it impacted policy at the Parliament*
- *Has it has assisted a lot in policy brief and a number of policies have been reviewed including the National Health Strategy and the National Health Insurance road map was developed.*
- *In terms of the HPSR priority topic sensitization among wider audience of stakeholders WHO ERA initiative played a crucial role. There is still undergoing discussions among health policy makers whether the policy options proposed through the evidence synthesis will be considered in health policy making.*
- *Policymakers have the option to conduct a more systematic search for evidence (which may or may not be more comprehensive compared to their current practice) before making a decision.*
- *Yes*
- *Yes*

### Helpful and least helpful aspects of WHO ERA initiative

**Q27. What did you find to be the most useful part of the WHO ERA initiative?**

- *What did you find to be the most useful part of the WHO ERA initiative?*
- *The it articulates the links of evidence and policy, identifies and trains relevant persons and follows them up for action is effective*
- *The fact that the Ministry was convinced to institutionalise such a platform in itself is a huge success and this formed a basis to support a much longer term plan to ensure routine and judicious use of evidence in policy and practice in the Ministry. As ZeipNET we even have plans to scale up the initiative to other Ministries*
- *The need for and use of evidence in informing and guiding policy deciion-making*
- *The knowledge and capacity of developing knowledge synthesis products have been increased. The demand from policymakers has been increased and they realized the usefulness of evidence-informed policymaking.*
- *Informed decisions at the top level*
- *The webinar sessions were so enlightening.*
- *The WHO ERA initiative was very well organized project and composition of its elements (learning platform, webinars, support on the additional requests) make this initiative successful and well accepted by the participants.*
- *Ongoing technical support.*
- *WHO ERA initiative is useful for me and my colleagues*

**Q28. What did you find to be the least useful part of the WHO ERA initiative?**

- *None*
- *I found some of the reporting a bit repetitive*
- *NA*
- *NA*
- *Everything was useful*
- *The on line lessons were not well participated by members, the practical engagement into the ERA activities provided more interest and knowledge.*
- *I have no answer to this question. For me, as a young researcher, every part of this initiative was very useful.*
- *Webinars - could be improved by a more open discussion between the countries on the challenges that they face.*
- *ERA helps us in division making process*

**Q29. Do you have any suggestions for how this initiative could be improved? This will improve future training initiatives that the Alliance for Health Policy and Systems Research supports.**

- *Not now*
- *As much as the idea in having the Ministry lead and control the budget in implementing this initiative is good especially in ensuring buy-in and sustainability, government bureaucracy and politics make it very difficult to make timely decisions and for timely implementation. I think there are other ways to ensure buy-in and sustainability while technical partners with experience in implementing projects lead.*
- *More interaction between the various platforms that will enhance learning and shared experiences.*
- *To encourage in-country teams to continue or start knowledge sharing about evidence-informed policymaking on a country or even regional level. To act as a national or regional hub in this direction*
- *Participation of team members should be done by all team members not only a few.Everyone should participate in the webinars.*
- *More in country workshops could be considered for more impact to the ERA platforms.*
- *More content on grey literature search. More content on sourcing online information systematically.*
- *The support should be for atleast 3 years. Not possible to build ecosystem of RES within one year only .*

**Q30. Are there any additional general comments/feedback you would like to provide?**

- *None*
- *Overally this was a great initiative and ZeipNET would even love to support other Ministries in the region to implement the same.*
- *None*
- *In general, the program is very interesting and important*
- *Thank you for your support and a good initiative*
- *I believe continued interaction with the ERA platforms in different countries and in country workshops including some once in a while webinars to keep the ERA platforms alive. This would provide continuous learning engagement to the ERA platform. Regional workshops for the countries who participated in the ERA could also continue to provide light on the ERA platforms.*
- *This is a very useful initiative that really supports evidence use in health policy making especially in low and middle income countries where evidence informed policy making is very limited.*
- *The modules that were taught to us were the mainstream rapid evidence synthesis methodology, however, after adapting the methodology we have found that in some countries, the evidence required may not be available from published articles.*

# Appendix 12: Evaluation and Feedback on ERA Components

*I. Summary of Online Systematic Reviews Course*

77 people were invited to the online course (5 - Georgia, 10 - India, 39 -Malaysia, 23 - Zimbabwe), and as of November 2019, 62 (81%) began the course, and 20 (26%) completed the course (*individual platform counts omitted for confidentiality*).

*II. Evaluation of Live Training Webinars*

Overall, 21 individuals (5 – Georgia, 4 – India, 7 – Malaysia, 5 – Zimbabwe) across the four LMIC platforms participated in one or more of the six live training webinars, with 10-16 per webinar. The number of participants who completed webinar evaluation surveys across the sessions ranged from 5-11, out of a roster of 22. Across the evaluated live webinars, most participants agreed or strongly agreed that presenters met session objectives (95.3%) and invited audience participation (95.7%). In terms of the technical aspects of the webinar presentations, most participants agreed or strongly agreed that the presentations were well-organized (95.3%) and provided enough time for interaction (86.3%). Audio and internet connectivity issues proved to be a consistent problem for some participants, which made it challenging for them to participate in the live sessions.

Throughout the webinars, participants identified that they liked the relevance and pragmatic discussion as well as opportunities to interact with experts and other platform participants. Opportunities to improve included having more time for topic presentation as well as questions and interaction during the webinar, increasing the number of real-time participants from each platform, and improving online connectivity. To address this feedback, webinars were increased from one hour to one-and-half hours, the number of allowed real time participants was increased from five members per platform to ten, and scheduled practice log-in/connection sessions with willing platforms were utilized to troubleshoot connectivity issues prior to the webinar presentations.

*III. Evaluation of in-country workshops*

Malaysia: 18 out of 26 participants completed the online evaluation form that asked them to rate the conduct of the sessions, relevance of the content, and what they liked, disliked, and learned from the training workshop. Overall, participants rated the sessions as good or important with an average score of 4.40 out of 5. All the items assessed were considered positive, with the lowest average score for any item being 4.30. All participants indicated that they would recommend the workshop to others.

Georgia: 17 out of 22 participants completed the assessment form, with an average score of 4.56 out of 5. Most strongly agreed that the material presented was applicable to their work and relevant to their professional development with an average score of 4.50 and 4.63, respectively. All the items assessed at the end of the workshop were considered positive with the lowest average score for any item being 4.44.

India: 14 out of 17 participants completed the assessment form, with an average score of 4.36 out of 5. Most strongly agreed that the material presented was applicable to their work and relevant to their professional development, with average scores of 4.64 and 4.86 respectively. All the items assessed at the end of the workshop were considered positive, and participants scored the workshops ability to enhance the capacity of the trainees to identify and characterize implementation strategies for each option considered the lowest at 4.21.

Zimbabwe: 10 out of 16 participants completed the assessment form, with an average score of 4.80 out of 5. Most strongly agreed that the material presented was applicable to their work and relevant to their professional development, with average scores of 4.90 and 5.00 respectively. Participants scored the workshop lowest on enhancing the capacity to characterize implementation considerations, with a score of 4.20.

Respondents emphasized that they liked that the in-country workshops were contextualized to the LMIC setting, practical examples were used, and concepts and processes related to the RR mechanism were clarified. They also expressed that in hindsight they would have benefited from more practical HPSR examples and content related to the LMIC context.

The TAC team created several avenues for cross-platform learning and support to take place. To create a more interactive and engaging environment, the TAC team made several attempts to facilitate cross-platform learning through discussion boards and live webinars. There was also dedicated discussion time at the end of every live webinar to allow for interaction between participants, the presenter, TAC team and each other. In addition, taking into consideration feedback received from the platforms throughout the initiative, the TAC team decided to use the last webinar as an opportunity for each platform to give a short presentation on their progress and anticipated impact, and obtain feedback from experts in HPSR and KS.

*IV. Summary of Platform Consultations and Online Support*

The Toronto TAC team offered a total of three consultation sessions with each of the ERA platform leads. The first consult had discussion questions sent to each of the ERA teams in advance of the call, in order to provide guidance on the discussion as well as facilitate tailored feedback. The focus of the first consult call was on generating demand, engaging stakeholders, and technical guidance on the process of rapid evidence synthesis. The second and third consultations were optional check-ins, intended to be participant-driven. All platforms except India participated in a second consult, and none of the platforms opted for a third consult.

The Toronto TAC team also provided ongoing support to all of the platforms via email (n=10) and the online discussion boards (n=5) on Canvas. Specifically, guidance, feedback, and resources on RES methodology (e.g. searching grey literature, quality appraisal, and screening of articles), reporting (e.g. reviewing draft RES products such as protocols and briefs), and stakeholder engagement (e.g. clarifying the research question) were provided.
