# Supplementary material for: Embedding rapid reviews in health policy and systems decision-making: Impacts and lessons learned from four low- and middle-income countries
Source: Health Res Policy Syst. 2023 Jun 6;21:45. doi: 10.1186/s12961-023-00992-w (PMC10243686; doi:10.1186/s12961-023-00992-w)
Supplement: Supplementary file 2 — Additional file 2. Tables S1–4. [file 12961_2023_992_MOESM2_ESM.docx]

**Table S1: Overview of the Selected LMIC Platforms**

| **Country** | **World Bank Economy Classification**  **2017*** | **Platform name, Location/Collaborator** | **Government/Ministry connection** | **Number of Core Members**** | **Number of Rapid Product Responses** | **Number of COVID-19 Products** |
| --- | --- | --- | --- | --- | --- | --- |
| **Georgia** | *Lower Middle-Income* | *ERA platform*, Curatio International Foundation (CIF) | Healthcare and Social Issues Committee (HSIC), Parliament of Georgia | 7 | 2 | 1 + additional requests |
| **India** | *Lower Middle-Income* | *Embedded Rapid Evidence Syntheses Unit (ERESU)*, The George Institute for Global Health, New Delhi | National Health Systems Resource Centre (NHSRC), Ministry of Health & Family Welfare (MoHFW) | 6 | 5 | 1 + additional requests |
| **Malaysia** | *Upper Middle-Income* | *Malaysian Alliance for Embedding Rapid Reviews in Health Systems Decision-Making (MAera)*, Institute for Health Systems Research, Kuala Lumpur | National Institutes of Health, Ministry of Health, Malaysia | 6 | 6 | 1 |
| **Zimbabwe** | *Low Income* | *Embedding Rapid Reviews in Health Systems Decision-Making: Zimbabwe (ERAZ),*  Zimbabwe Evidence Informed Policy Network (ZeipNET) | Ministry of Health and Child Care, Zimbabwe | 7 | 3 | 2 |
| ** For the 2017 fiscal year, low-income economies were defined as those with a GNI per capita, calculated using the World Bank Atlas method, of $995 or less; lower middle-income economies are those with a GNI per capita between $996 and $3,895; upper middle-income economies are those with a GNI per capita between $3,896 and $12,055; high-income economies are those with a GNI per capita of $12,055 or more.*  *** “Core” as inferred from the LMIC progress updates (monitoring forms) submitted to the Technical Assistance Centre. “Core” reflects the research-related “pool”, comprising internal government staff, and external expertise (e.g. Curatio) where identified. It does not reflect FTE (full-time equivalent) staffing.* | | | | | | |

**Table S1B: Key Dates**

| **Country** | **Platform name, Location** | **Webinar #1** | **Platform Team in Place** | **In-Country Workshop** | **Start of First Rapid Review** | **Webinar #6** | **Comments** |
| --- | --- | --- | --- | --- | --- | --- | --- |
| **Georgia** | *ERA platform*, Curatio International Foundation (CIF) | Feb 2019 | Mar 2019 | May 2019 | July 2019 | Sep 2019 |  |
| **India** | *Embedded Rapid Evidence Syntheses Unit (ERESU)*, The George Institute for Global Health, New Delhi |  | Mar 2019  (Oct 2019) | June 2019 | Mar 2019 |  | Recruitment challenges described |
| **Malaysia** | *Malaysian Alliance for Embedding Rapid Reviews in Health Systems Decision-Making (MAera)*, Institute for Health Systems Research, Kuala Lumpur |  | Oct 2018 | Nov 2018 | Nov 2018 |  | Recruitment and retention challenges described |
| **Zimbabwe** | *Embedding Rapid Reviews in Health Systems Decision-Making: Zimbabwe (ERAZ),*  Ministry of Health and Child Care, Harare |  | May 2019 | June 2019 | July 2019 |  |  |
| *TAC = Technical Assistance Centre. ERA = Embedded Rapid Reviews* *in Health Systems Decision-Making*  *Dates are based on WHO TAC ERA monitoring reports.* | | | | | | | |

**Table S2: LMIC Platform Requests and Rapid Responses**

|  | **Type of Question*** | **Issue** | **Rapid Review Focus** | **Product Title** | **Impacts / Downstream Targets** |
| --- | --- | --- | --- | --- | --- |
| Georgia | Health Financing | The Health and Social Issues Committee (HSISC) recognized that access to pharmaceuticals is challenged by high out of pocket expenses. | Pricing and purchasing policies regarding access to pharmaceuticals. | *Pharmaceutical pricing policies to improve the population’s access to pharmaceuticals in Georgia* | National policy discussion on regulatory changes. Project ongoing. |
|  | Health Financing | The healthcare system is highly privatised, with few incentives for quality improvement. The HSISC is interested in pay-for-performance, amidst a lack of consensus among policymakers. | Effects of pay-for-performance on quality improvement in primary healthcare. | *Effects of Pay for Performance on utilization and quality of care among Primary Health Care providers in Middle and High-Income countries* | To be determined. Project ongoing. |
|  | Health Service Delivery (COVID-19) | MoILHSA requested evidence worldwide for approaches to limiting transmission. | Epidemic containment measures and consequences; epidemic modelling; Rt estimates | *The COVID-19 epidemic in Georgia Projections and Policy Options* | Policy recommendations, modelling for Prime Minister’s office, including lockdown decisions |
| India | Health Workforce | India's CPHC programme is placing emphasis on mid-level providers for primary health care delivery at Health and Wellness Centres. | Effectiveness of mid-level providers corresponding to the 12 CPHC service packages. | *Mid-Level Health Providers (MLHPs) for Primary Healthcare: Rapid Policy Brief* | National and state-level consultation on policy for mid-level providers. |
|  | Health Service Delivery | State resource constraints have motivated interest in ‘Angle of Tri-Radius (ATD) measurement’ as an alternative to traditional breast cancer screening by frontline workers. | Examine diagnostic accuracy and cost-effectiveness of ATD measurement, as well as barriers and enablers. | *Palmar Angle of Tri-Radius for Breast Cancer Screening in Women* | Policy on use of ATD measurement for breast cancer screening in resource-poor settings. |
|  | Health Service Delivery | The state would like to improve access to dialysis programmes so that chronic kidney disease patients have better health outcomes. | Health systems interventions that improve dialysis outcomes. | *Health Systems Interventions to Improve Dialysis Outcomes in Patients with Chronic Kidney Disease on Hemodialysis* | To be determined. Project ongoing. |
|  | Health Workforce | The District Medical Officer plans to develop training modules to address harmful post-natal care practices. | 1. Postnatal care best practices.  2. Postnatal harmful practices and implications for LMICs.  3. Best practice for development of training modules. | *Training of post-natal care attendants for post-natal care, nutrition and breastfeeding: rapid policy brief* | Training modules for post-natal care attendants. Ongoing. Delayed due to COVID-19. |
|  | Health Service Delivery | The state has identified a high burden of asthma and COPD, and desires evidence-informed policy in an LMIC context. | 1. Diagnosis of COPD  2. Quality of Care in children with asthma and adults with COPD  3. Primary prevention of asthma and COPD | 1. *Accuracy of Screening Tests for Chronic Obstructive Pulmonary Disease in Primary Health Care: Rapid evidence synthesis*  2. *Interventions to improve quality of care in children and adolescents with asthma in primary health care settings: rapid policy brief*  3. *Primary prevention of asthma and chronic obstructive pulmonary disease at the primary healthcare level: rapid policy brief* | State-level policy discussion and formation. Project ongoing. Delayed due to COVID-19. |
|  | Health Workforce (COVID-19) | The National Health Systems Resource Centre (NHSRC) wanted to prepare community front line health workers (FLHWs) for prevention and control of COVID-19 | 1. Roles, issues, barriers, and enablers for FLHWs for prevention and control  2. Resources for guidance, training manuals and information for FLHWs | *Frontline health workers in COVID-19 prevention and control: rapid evidence synthesis*, and an inventory of guidelines and training materials for FLHWs | Brochure, posters for India's community health workers; Odisha government policy, including addition of field surveillance, and more. |
| Malaysia | Health Workforce | Reducing the maternal mortality rate is complicated by increasing complex maternal cases. The Ministry of Health (MoH) wants to prepare the workforce accordingly. | Qualifications for healthcare practitioners managing antenatal cases in primary care, with initial focus on midwives | *Midwifery Qualification in Primary Care* | Pilot degree-level education requirements for nurses |
|  | Health Service Delivery | The MoH, Nursing Division, noted outcome measures for pressure injury were not comparable with other countries. | Outcome indicators for pressure injury. | *Outcome Indicators for Monitoring of Pressure Injury Prevention - Report* | National guideline with updated definition and outcome indicators |
|  | Health Service Delivery | The Traditional and Complementary Medicine (T&CM) Division would like to optimise the integration of T&CM services into the national healthcare system | Models for integrative medicine | *A Rapid Review on Approaches for Integrative Medicine* | Strategic plan for the T&CM Division (aligned with the WHO Traditional Medicine Strategy 2014-2023) |
|  | Health Service Delivery | The Public Health Development Division was interested in expanding healthcare policies to include well-being in the 12^th^ Malaysia Plan. | Frameworks for including population well-being outcomes in population health plans | *Rapid Evidence Inventory – Population Well-being Outcomes Framework* | The national 12^th^ Malaysia (5-year) Plan |
|  | Health Service Delivery | A hospital using telemedicine services for infectious diseases was interested in patient experiences related to telemedicine | Assessment tools related to patient satisfaction in telemedicine | *Patient Satisfaction Tools in Telemedicine – A MAera Rapid Evidence Inventory* | Inventory of satisfaction assessment tools for use in telemedicine patients |
|  | Health Service Delivery | The National Head of Service for Internal Medicine sought to enhance multidisciplinary integration between teams managing patients with complex multimorbidity. | Methods to assess care integration | *Integrated Care Measurement* | To be determined. Project ongoing. Delayed due to COVID-19. |
|  | Leadership & Governance (COVID-19) | The Deputy Director General requested a summary of Malaysia's health systems response to COVID-19 for public dissemination | Responses and strategies for COVID-19 between 1 Jan 2020 - 17 April 2020 | *Online dashboard, report pending* | Online public dashboard of government responses to COVID-19 and journal publication |
| Zimbabwe | Health Financing | The Ministry of Health and Child Care (MoHCC) was interested in a sustainable health financing framework for Universal Health Coverage. | Enrolment of informal sector into national health insurance. Enrollment criteria/ guideline for the informal sector. | *Rapid Review on enrolment of informal sector into the National Health Insurance. Enrollment Criteria/ guideline for the informal sector.* | Cabinet paper, bill on national health insurance |
|  | Health Financing | The Zimbabwe Statistical Agency and the Ministry of Health were seeking to optimize health expenditure for the universal health coverage program. | International standardised tools for collecting, analysing, and describing health financing systems, and system for Health Accounts | *Policy Brief on the National Health Accounts* | Health Financing Symposium. Ongoing. |
|  | Health Service Delivery | Ministry of Health and Child Care, HIV and AIDS unit wanted to improve uptake of HIV self -testing | Barriers and facilitators to uptake of HIV self-testing | *Rapid review on the factors affecting the uptake of HIV Self Testing among adults in Zimbabwe.* | Re-orientation of the current HIV self testing program. Ongoing. |
|  | Health Service Delivery (COVID-19) | National Inter-ministerial Task Force on Covid 19 needed to recommend the duration of institutional quarantine for returning citizens | Ideal duration of institutional quarantine, and requirements for success | *Rapid review on mandatory institutional quarantine for returning residents.* | National policy on quarantine for returning citizens |
|  | Health Service Delivery (COVID-19) | National Inter-ministerial Task Force on Covid 19 wanted evidence on implementation of face masks, as lockdowns were being reduced | Effectiveness and considerations for use of face masks. | *Rapid review on the effectiveness of use of non-surgical face masks by the public/community for effective prevention and control of COVID 19, in low resource settings* | National policy on face masks |
| *Based in the WHO Health Systems Building Blocks framework. | | | | | |

**Table S3: Platform-Identified Barriers and Facilitators to Knowledge User Engagement**

|  | **Issue** | **Barriers** | **Facilitators** |
| --- | --- | --- | --- |
| Georgia | Access to pharmaceuticals | The review question was broad, making it challenging to narrow the original research question. As such, the time taken to reach consensus was longer than expected.  Busy political environment linked with election policy formulation reduced policymakers’ availability for meetings. | Increased interest of government in pharmaceutical access, which attracted wider attention. |
|  | Pay-for-performance | There was low engagement (40% response rate) in the prioritization exercise. |  |
|  | Limiting COVID-19 transmission |  |  |
| India | Mid-level providers | The mid-level health provider position is being operationalized in varying ways, which does not always match international definitions. This was a challenge in explaining our findings to stakeholders.  Differing capacity of stakeholders. | Facilitators that enabled successful engagement included topic awareness, mapping and getting the right stakeholders, and establishing partnership with knowledge users early in the process |
|  | Angle of Tri-Radius (ATD) measurement | The only barrier encountered was the clarification of the topic and the question to be addressed in terms of its utility. | The inclusion criteria and search strategy were agreed at an early stage within a short time.  Engaging content experts and continuously engaging requesters helped clarify the review question and understand diagnostic utility. |
|  | Kidney Dialysis Programmes | There was a slight delay in coming to consensus on the review question and agreeing upon the inclusion criteria. |  |
|  | Post-Natal Care Practices | There were delays in protocol approval as the requester was busy and on leave for the holidays. |  |
|  | Burden of asthma and COPD |  |  |
|  | Frontline COVID-19 Health Workers |  | We were able to generate the output within a weekend, which ensured the material could be used. |
| Malaysia | Complex Maternal Cases | Little participation from knowledge users in review conduct. | The discussion was time-consuming as the main stakeholder wanted to include others. A non-disclosure agreement was introduced to allow Involvement beyond MOH.  Ensured shared goals amongst different knowledge users. Importance of clarity of review question and use of gray literature.  Policymakers required platform assistance to translate findings. |
|  | Pressure Injury | It took some time to finalize the methodology, as the core team had limited experience conducting reviews.  Commitments of external team members (decision makers). It was an additional task for them. Two trained research assistants resigned. | Availability of technical advice from TAC, consultation with academic partners who were systematic review methodology experts.  Commissioning policymakers nominated other stakeholders who should be involved in the final presentation. This facilitated fruitful discussion during the meeting, resulting in immediate decision-making for pressure injury monitoring. |
|  | Traditional and Complementary Medicine (T&CM) Services | Stakeholders were unable to meet the core team frequently within a short timeframe due to competing priorities. | When a face-to-face discussion with stakeholders officers was not possible, phone enabled prompt clarification of issues. Stakeholders responded promptly to email.  Support from the Director of T&CM Division was a facilitator. |
|  | 12^th^ Annual Malaysia Plan -Well-being | Approval for conducting review for an urgent request was a challenge. | The stakeholders were forthcoming on the idea of a virtual discussion to ensure timely communication. |
|  | Telemedicine experiences |  | *see Hospital Telemedicine services* |
|  | Multidisciplinary integration in multimorbidity |  | Grouping findings into themes or mapping against a framework helped the stakeholder’s understanding. |
|  | Malaysia's COVID-19 response |  |  |
| Zimbabwe | Universal Health Coverage | The time taken to get the draft product was longer than anticipated due to other competing ERAZ team commitments. | Buy-in from the Cabinet and other key line ministries which made it less of a challenge for the ERAZ team to engage key stakeholders.  A rapid review approach was agreed with a negotiated timeline, inclusion criteria, and search strategy. |
|  | Universal Health Coverage - Health Information |  | Product had a lot of stakeholder support because of capacity development activities with other partners in health financing. |
|  | Uptake of HIV self -testing | The COVID-19 pandemic delayed project completion. |  |
|  | Covid 19 quarantine for returning citizens |  | Platform made “extra” efforts to help in actual use of the evidence synthesized to produce the policy document, recognizing ministry was overwhelmed. Engaging the College of Public Health Physicians strengthened networks and set the tone for the use in policymaking.  Platform embedded within the Ministry of Health enabled policymaker engagement. |
|  | Covid 19 - Face Masks |  | One big lesson is embedding the platform among the policymakers. Product had a lot of stakeholder support because of capacity development activities with other partners in health financing. |
| *Responses have been edited for clarity and brevity. Responses based on reports of ‘facilitators and barriers’ and ‘lessons learned’.* | | | |

**Table S4: Challenges and Lessons Learned (as assessed by the TAC)**

| **Challenges and Lessons Learned** |
| --- |
| *Context-specific expertise*  Although one of the TAC centers was based out of Uganda, platforms might have benefited from more support from health policy and systems research (HPSR) experts based in other LMICs. Commonly encountered evidence synthesis methods are tuned to clinical topics, while HPSR rapid evidence is inherently more complex, particularly so when applied to unique LMIC settings. Such expertise should bring to bear parallel LMIC HPSR examples, highlighting lessons learned from previous efforts. In addition to the platform-strategic SWOT analysis at the outset, a country-level mapping of capacities may be useful for identifying such expertise before searching elsewhere. We also suggest leveraging the experience of established platforms that have previously benefited from similar initiatives. This could also foster a community of practice of benefit to emerging platforms. |
| *Cross-platform learning*  The opportunities provided through cross-platform learning are articulated in education and online learning theory [1-6] and were highly desired by platforms. Despite the opportunities provided for cross-platform learning, more could have been done to support inclusive, interactive learning to facilitate meaningful cross-platform engagement. Participant feedback indicated more webinar time could have been allowed for questions and discussion, and TAC-generated engagement questions could have been posted and managed through the cross-platform CANVAS portal. As well, feedback was regularly collected on an excel form. Changing the format to an online discussion between the platforms could encourage more interaction. We recommend proactively establishing formal expectations for cross-platform learning from the start. This can be done is through online meetings prior to the official launch of the initiative, structured discussion topics, and provision of presentation templates. “Meet-and-greet” sessions are also a low-stakes approach for creating relationships, establishing trust, and building rapport. A shared database of HPSR evidence syntheses might also be considered.  All four teams were at different stages of platform establishment at inception. In general, some teams might already have projects early on compared to other teams, and this may influence the degree and kind of support required. This could also make cross-platform learning challenging. |
| *Sustainability*  Throughout this initiative, all platforms were encouraged to build relationships with their knowledge users and establish buy-in for rapid evidence synthesis products. When the COVID-19 pandemic hit, platforms were better positioned to respond to demand for rapid COVID-19-related reviews. Given the success of having platforms create and implement their own start-up engagement plans, we recommend similar planning with sustainability of the platform in mind.  Another aspect of the initiative that remains paramount is the requirement of a partnership with a governmental organization with (likely) rapid evidence needs, that can provide resources/support, and champion rapid evidence synthesis for decision-making. An existing long-term relationship and target of dedicated budget line for a rapid evidence team are perhaps ideal. Identifying ministry budget line items and aligning research accordingly might also be something to consider. Once credibility is established, platforms might even look beyond sustainability to growing these services within other departments and organizations.  Many platforms indicated they could benefit from ongoing networking opportunities and collaboration with other groups in similar contexts. In this way, a sustainable system of platform support could be established and maintained by interested organizations. Sustaining this community of practice could not only provide ongoing support to existing platforms but could also encourage these platforms to autonomously scale-up their systems and systematically spread these across regions, countries, and even globally. In this situation, collective LMIC expertise could be leveraged to support ongoing LMIC health systems improvements. This community of practice could be established through online networks, where the platforms can connect and continue to support the development and maintenance of their work well after the official initiative has ended. |
